# Survival trends in gastric cancer patients between 1987 and 2016: a population-based cohort study in Finland

**DOI:** 10.1007/s10120-022-01326-5

**Published:** 2022-08-07

**Authors:** Urgena Maharjan, Joonas H. Kauppila

**Affiliations:** 1 Present Address: Surgery Research Unit, Medical Research Center Oulu, Oulu University Hospital and University of Oulu, Oulu, Finland; 2 Upper Gastrointestinal Surgery, Department of Molecular Medicine and Surgery, Karolinska Institutet and Karolinska University Hospital, Stockholm, Sweden

**Keywords:** Comorbidity, Stomach neoplasms, Prognosis, General surgery, Survival rate

## Abstract

**Background:**

Gastric cancer is the fourth leading cause of cancer-related deaths globally. There is a paucity of national studies examining gastric cancer mortality in relation to treatment status. This study evaluated the survival trends in gastric adenocarcinoma and all gastric cancers stratified by treatment in Finland during 1987–2016.

**Methods:**

This population-based, nationwide, retrospective cohort study included all gastric cancer patients registered in the Finnish Cancer Registry and Patient Registry. The survival rates were calculated for 1, 3, and 5 years, stratified by treatment. Prognostic factors were determined using Cox regression.

**Results:**

A total of 18,713 non-cardia gastric adenocarcinoma, and 3617 cardia adenocarcinoma patients were included. Surgical treatment decreased for non-cardia adenocarcinoma and remained constant for cardia adenocarcinoma. In non-cardia adenocarcinoma, the 5-year survival declined from 17% to 16% from 1987–1991 to 2012–2016. In surgically treated patients, survival increased from 29% to 38%, while an increase from 4% to 7% in those undergoing chemotherapy and decrease from 6% to 3% in those not receiving any treatment were observed. In cardia adenocarcinoma, the 5-year survival increased from 10% to 18% in all patients, 16% to 40% in surgical patients, 0% to 5% in patients receiving chemotherapy, and from 5% to 9% in patients receiving no treatment. Earlier calendar periods, older age, male sex, and higher comorbidity were risk factors for poor prognosis.

**Conclusions:**

Gastric non-cardia adenocarcinoma survival declined, limited to advanced stage patients not receiving any treatment. Gastric cardia cancer survival seems to have improved over time in Finland.

**Mini abstract:**

This study evaluated survival trends of gastric cancer in Finland during 1987–2016 and established that the 5-year survival is declining in non-cardia adenocarcinoma but improving in all gastric cancers.

**Supplementary Information:**

The online version contains supplementary material available at 10.1007/s10120-022-01326-5.

## Introduction

Gastric cancer is the fifth most common cancer and the fourth leading cause of cancer-related death around the world [1]. Gastric cancers can be divided anatomically into non-cardia and cardia cancer, and histologically into intestinal and diffuse types [2]. Gastric non-cardia and cardia cancers exhibit differences in their etiology, incidence, and prognosis [2–4]. The incidence of gastric cardia cancer is increasing along with increasing obesity and reflux, while non-cardia cancer incidence is decreasing due to the lower prevalence of *Helicobacter pylori* infection [5–7]. Prevention of colonization of *H. pylori* has shown a potential to reduce incidences of gastric cancer [3, 8], but the best curative treatment for gastric cancer patients includes early diagnosis and surgical resection [9].

Although gastric cancer incidence is declining and survival is improving in most countries, the survival of patients after curative surgery is poor. In Europe, despite the decline in incidence and improvement in survival, gastric cancer remains a highly fatal disease with a poor prognosis. The 5-year survival rate of gastric cancer in Europe fluctuates between 10% and 30% [10–12]. In Sweden, the survival after non-cardia gastric cancer diagnosis did not improve over 20 years [13]. The survival trends of gastric cancer in Finland are not known. Therefore, this study aimed to evaluate the survival trends of gastric cancer stratified by location and treatment in Finland during 1987–2016.

## Materials and methods

### Study design

This study was a population-based, nationwide, retrospective cohort study of all gastric cancer patients identified in Finland during the period 1987–2016, based on Finnish national registries. The study was approved by the Northern Ostrobothnia ethical committee (EETMK 115/2016) and relevant governmental bodies. Informed consent was not required for registry research [14, 15].

### Data sources

All patients with gastric cancer were identified from the Finnish Cancer registry (FCR) and the Finnish Patient Registry (HILMO) using respective ICD-9 (151) and ICD-10 (C16) codes. Personal identification numbers assigned to all residents in Finland were used to combine registry data. The FCR provided data on incident cancers, date of diagnosis, cancer histology type, location, cancer stage, as well as the age of patients at diagnosis and sex of patients. FCR also provides data on whether patients had chemotherapy. HILMO was used to identify incident cancers and patients undergoing surgical resection and to calculate comorbidity in all identified gastric cancer patients, as previously defined. Surgical codes from the patient registry helped identify surgical procedure types which included gastrectomy, esophagectomy, esophagogastrectomy, endoscopic mucosal resection (EMR), and endoscopic submucosal dissection (ESD). Both open and laparoscopic surgery was included [14, 15]. FCR and HILMO are comprehensive registries in Finland, since physicians are mandated to report all incident cancers to FCR, and all healthcare units are obligated to report diagnosis and operations codes to HILMO. Thus, these operation codes serve as the basis for reimbursement [16, 17]. The Finnish death registry provided dates of death (until December 31, 2019) and cause of death (until December 31, 2018). The data in the Finnish death registry are 100% complete for vital status [18].

### Data analysis

All analyses were based on a prior study protocol. Data on all patients diagnosed with gastric cancer from 1987 to 2016 were retrieved from FCR and HILMO registries. The patients were then grouped according to 5-year periods (1987–1991, 1992–1996…), age groups (< 50, 50–59, 60–69, 70–79, 80–89 and > 90), sex, Charlson Comorbidity Index score (0, 1, 2, 3 +), surgery status (yes and no), chemotherapy (no, yes, and missing), and stage (local, locally advanced, and advanced). For survival analysis, 1-year, 3-year, and 5-year survival rates were calculated for both non-cardia and cardia gastric adenocarcinoma separately, as well as stratified further by treatment: surgery, or no surgery; surgery, chemotherapy only, or no treatment; as well as by cancer stage: local, locally advanced, or advanced. Survival analysis was conducted using the life table method and plotted using Kaplan–Meier curves. Cox regression was used to calculate hazard ratios (HR) of mortality with 95% confidence intervals (95% CI) for each stratified variable (calendar year, age group, sex, and comorbidity score) which are considered prognostic factors associated with gastric cancer mortality. Finally, a separate analysis for all gastric cancer types was done to evaluate whether the survival trends were similar to trends of gastric adenocarcinoma. Analyses were carried out using IBM SPSS 26 (Armonk, NY, USA).

## Results

### Patients

Of the total 22,330 gastric cancer patients, 18,713 (83.8%) had non-cardia cancer and 3617 (16.2%) had cardia cancer during 1987–2016. After exclusion of 797 neuroendocrine tumors, 518 other types of tumors such as squamous cell carcinoma, melanoma, mesenchymal cancer, and gastrointestinal stromal tumor, and 3275 patients without any information on histology available, there were 18,713 histologically confirmed gastric non-cardia adenocarcinomas, and 3617 gastric cardia adenocarcinomas included in the analysis. The number of both cardia and non-cardia adenocarcinoma patients decreased over time. Most gastric adenocarcinomas occurred at the age of 70–79 years. There were more men with gastric non-cardia (52.6%) and cardia adenocarcinoma (70.0%) than women. Most gastric adenocarcinoma patients did not undergo chemotherapy and the majority had advanced cancer stages (Table 1). The demographics in all gastric cancer types were highly similar to histologically confirmed gastric adenocarcinomas. The number of gastric cardia cancer of any histology, however, remained constant over time (Supplementary Table 1).

**Table 1:** Histologically confirmed gastric non-cardia and cardia adenocarcinoma patients stratified by calendar period, age, sex, CCI, surgical treatment, chemotherapy, and cancer stage

Variable	Gastric non-cardia adenocarcinoma *n* (%)	Gastric cardia adenocarcinoma *n* (%)	Total *n* (%)
Total	18,713 (83.8)	3617 (16.2)	22,330 (100)
Calendar period			
1987–1991	4486 (24.0)	677 (18.7)	5163 (23.1)
1992–1996	3720 (19.9)	676 (18.7)	4396 (19.7)
1997–2001	3135 (16.8)	657 (18.2)	3792 (17.0)
2002–2006	2928 (15.6)	551 (15.2)	3479 (15.6)
2007–2011	2552 (13.6)	612 (16.9)	3164 (14.2)
2012–2016	1892 (10.1)	444 (12.3)	2336 (10.5)
Age			
> 50	1325 (7.1)	265 (7.3)	1590 (7.1)
50–59	2243 (12.0)	523 (14.5)	2766 (12.4)
60–69	4197 (22.4)	929 (25.7)	5126 (23.0)
70–79	5910 (31.6)	1126 (31.1)	7036 (31.5)
80–89	4398 (23.5)	678 (18.7)	5076 (22.7)
90 +	640 (3.4)	96 (2.7)	736 (3.3)
Sex			
Female	8864 (47.4)	1085 (30.0)	9949 (44.6)
Male	9849 (52.6)	2532 (70.0)	12,381 (55.4)
CCI			
0	12,007 (64.2)	2222 (61.4)	14,229 (63.7)
1	4058 (21.7)	817 (22.6)	4875 (21.8)
2	1717 (9.2)	375 (10.4)	2092 (9.4)
3 +	931 (5.0)	203 (5.6)	1134 (5.1)
Surgery			
Yes	8293 (44.3)	1320 (36.5)	9613 (43.0)
No	10,420 (55.7)	2297 (63.5)	12,717 (57.0)
Chemotherapy			
No	12,909 (69.0)	2255 (62.3)	15,164 (67.9)
Yes	2758 (14.7)	694 (19.2)	3452 (15.5)
Missing	3046 (16.3)	668 (18.5)	3714 (16.6)
Stage			
Local	3608 (19.3)	599 (16.6)	4207 (18.8)
Locally advanced	2226 (11.9)	469 (13.0)	2695 (12.1)
Advanced	7610 (40.7)	1452 (40.1)	9062 (40.6)
Unclear or missing	5269 (28.2)	1097 (30.3)	6366 (28.5)

*CCI* Charlson Comorbidity Index

### Surgery

**Fig. 1:**
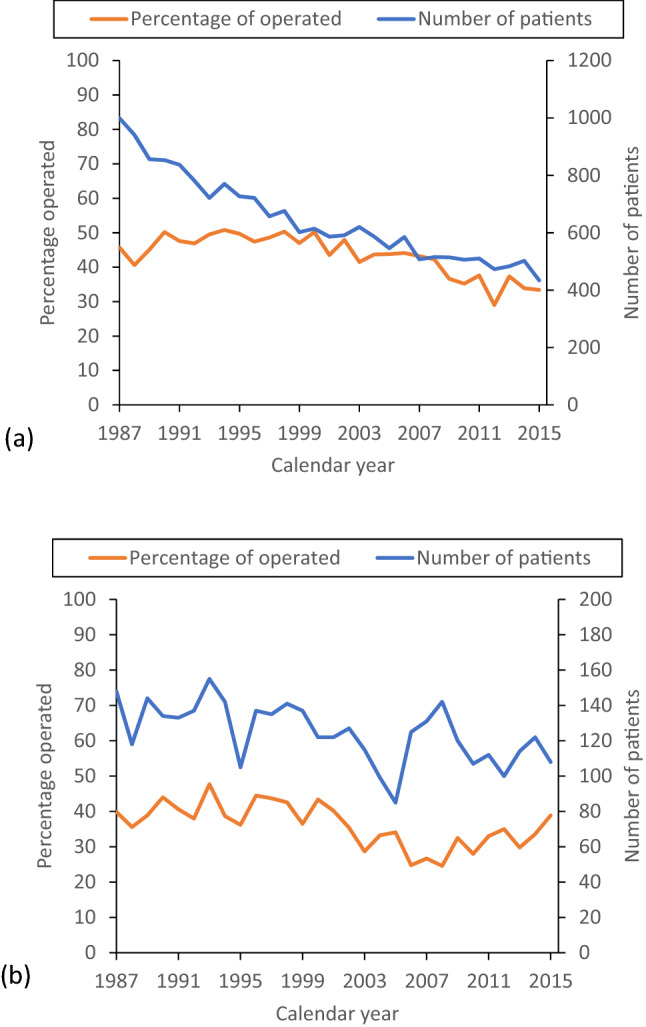
Graphs showing number of histologically confirmed gastric non-cardia adenocarcinoma** a** and gastric cardia adenocarcinoma **b** patients diagnosed in Finland between 1987 and 2016. The curves show the number of patients diagnosed (blue line) and proportion of patients undergoing surgery (orange line)

A total of 9613 (43.0%) gastric adenocarcinoma patients underwent surgical treatment. For gastric non-cardia adenocarcinoma, the proportion of surgical treatment was larger 8293 (44.3%) than for gastric cardia adenocarcinoma (1320, 36.5%, Table 1). The proportion of patients who had undergone surgery decreased from 45.0% to 34.0% for gastric non-cardia adenocarcinoma (Fig. 1a), but remained constant for gastric cardia adenocarcinoma between 1987 and 2016 (Fig. 1b). In the separate analysis of gastric cancers of any histology, the proportion of patients who underwent surgery declined in both gastric cardia and non-cardia cancers (Supplementary Fig. 1).

### Survival trends in gastric non-cardia cancer

#### All patients

**Fig. 2:**
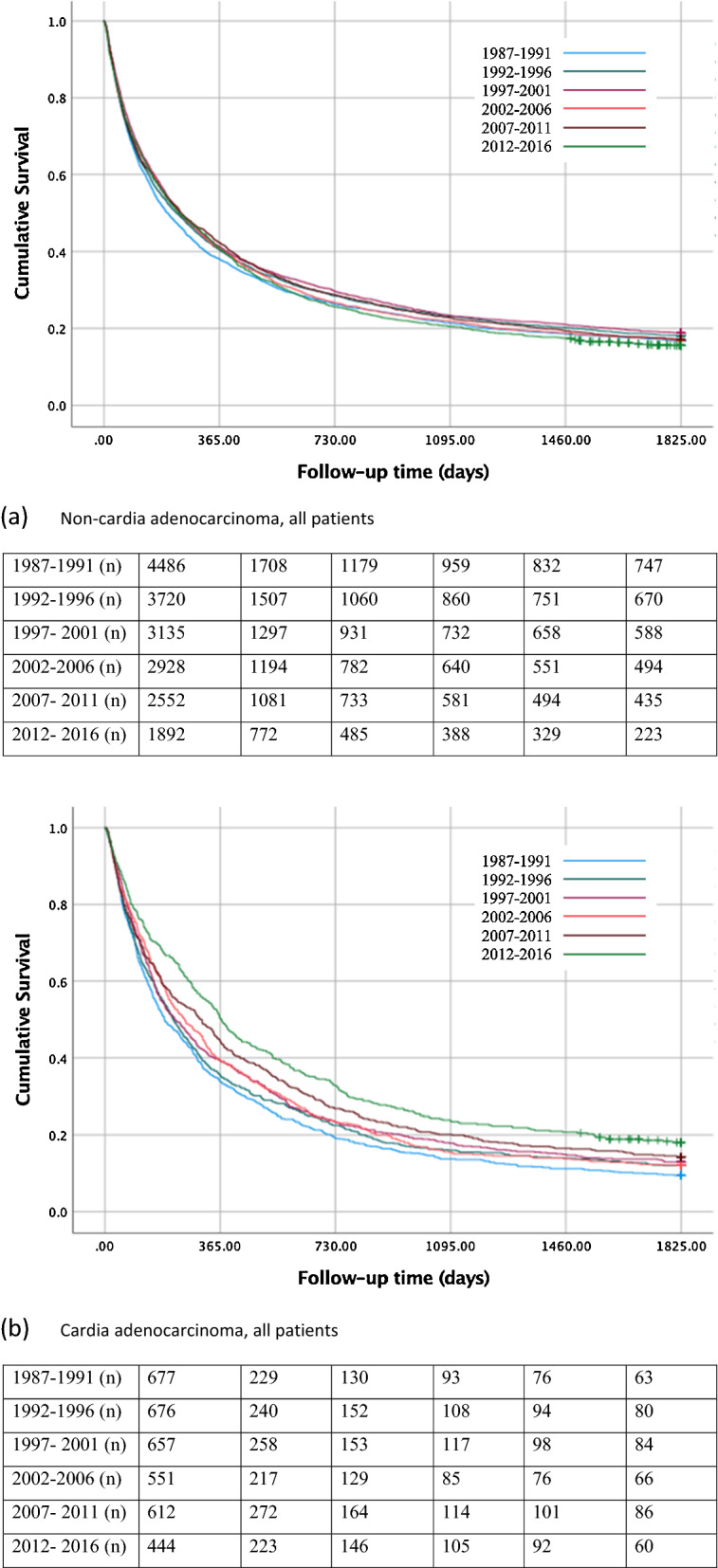

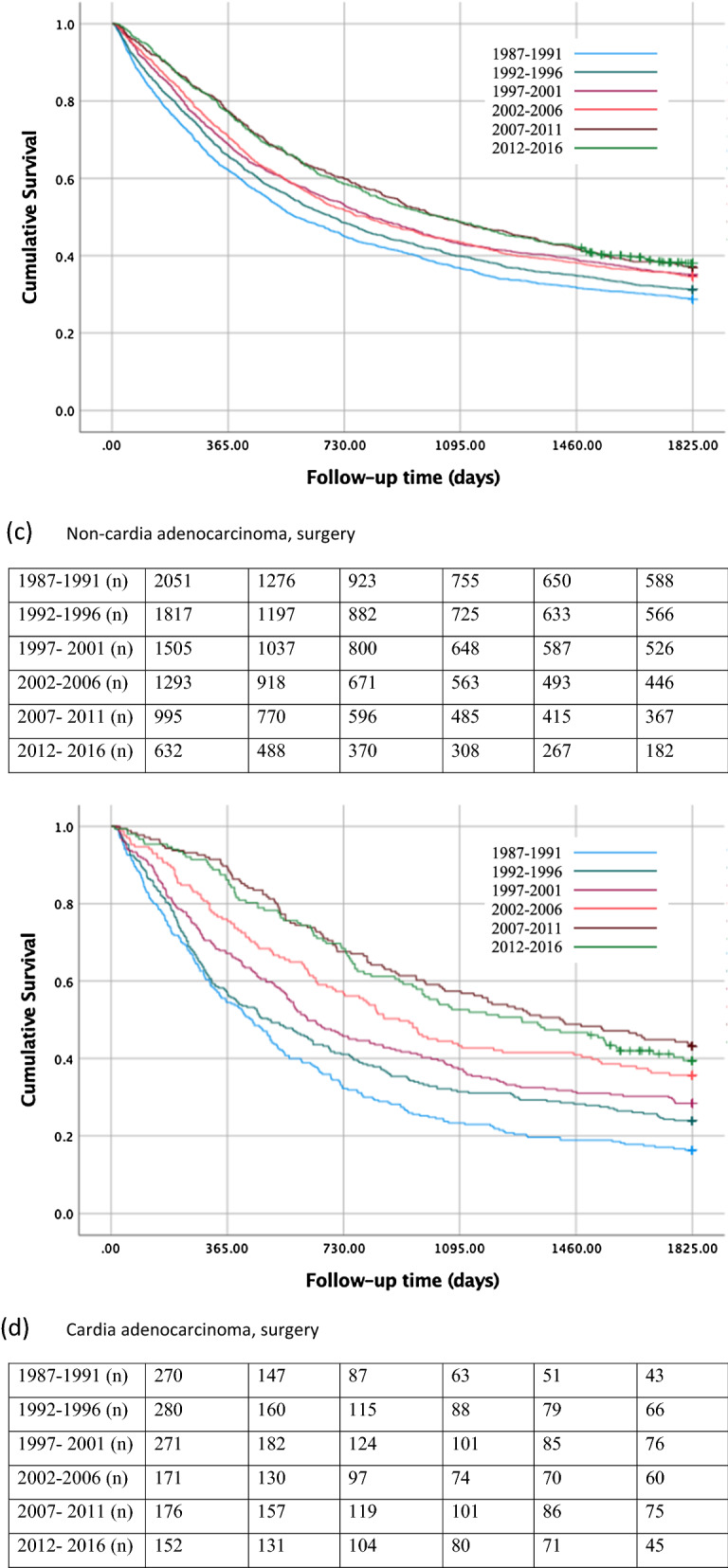

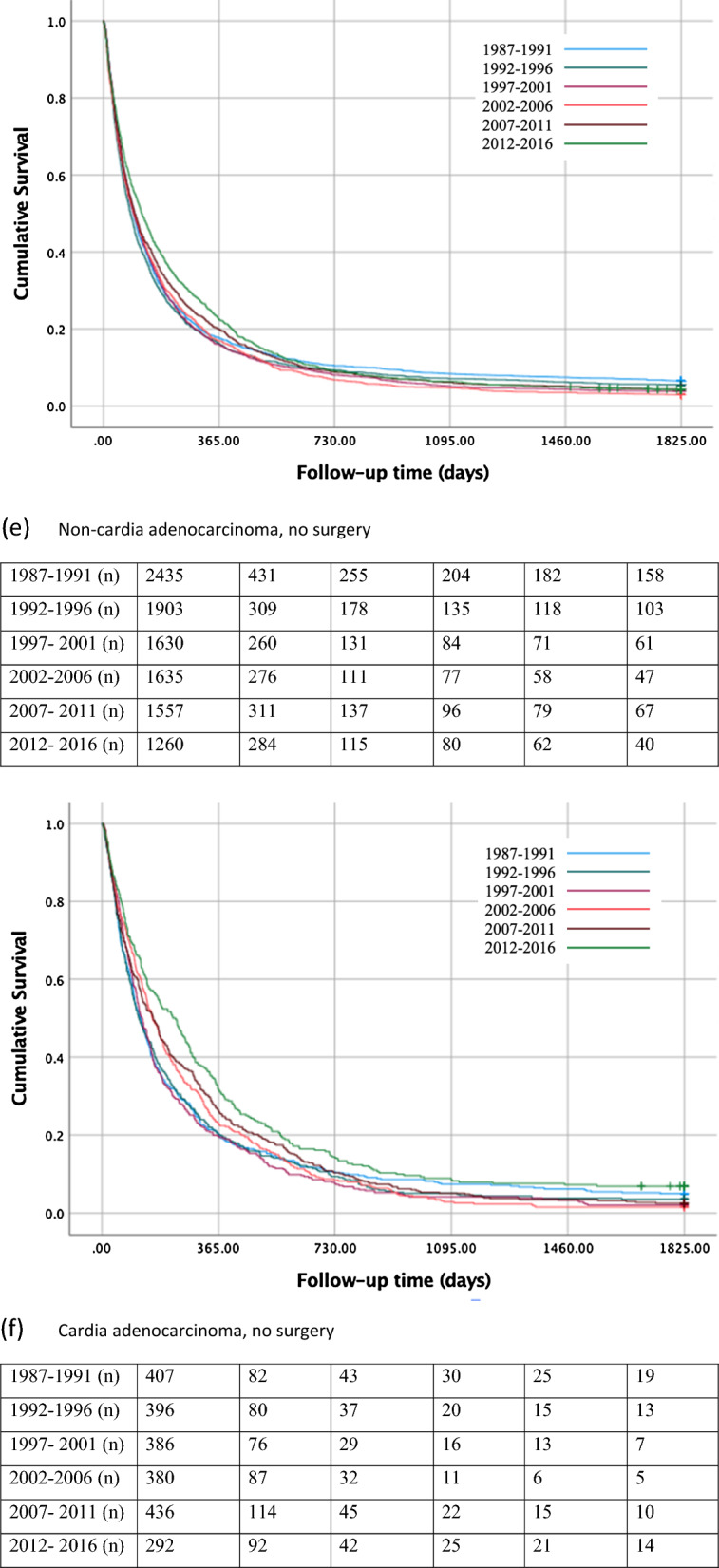
Kaplan–Meier survival curves showing 5-year survival rate for histologically confirmed gastric non-cardia adenocarcinoma **a** and gastric cardia adenocarcinoma** b** in all patients, for gastric non-cardia adenocarcinoma** c** and gastric cardia adenocarcinoma **d** undergoing surgery, and for gastric non-cardia adenocarcinoma **e** and gastric cardia adenocarcinoma** f** not undergoing surgery, stratified by 5-year time periods

In all patients with gastric non-cardia adenocarcinoma, the 5-year survival rate decreased from 17% during 1987–1991 to 16% in 2012–2016 (Table 2; Fig. 2a). For gastric cancer patients of any histology, the 5-year survival rate increased from 19% during 1987–1991 to 24% during 2012–2016 (Supplementary Table 2, Supplementary Fig. 2a).

**Table 2:** One- year-, 3-year-, and 5-year survival in histologically confirmed gastric adenocarcinoma (non-cardia and cardia) stratified by treatment during 1987–2016

	Gastric non- cardia adenocarcinoma				Gastric cardia adenocarcinoma			
	Patients	Survival in %			Patients	Survival in %		
Calendar period	Number (%)	1 year	3 years	5 years	Number (%)	1 year	3 years	5 years
All patients								
1987–1991	4486 (24.0)	38	21	17	677 (18.7)	34	14	10
1992–1996	3720 (19.9)	40	20	18	676 (18.7)	36	16	12
1997–2001	3135 (16.8)	41	23	19	657 (18.2)	40	18	13
2002–2006	2928 (15.6)	41	22	17	551 (15.2)	40	16	12
2007–2011	2552 (13.6)	42	23	17	612 (16.9)	44	20	14
2012–2016	1892 (10.1)	40	23	16	444 (12.3)	50	24	18
Surgery								
1987–1991	2051 (24.7)	62	37	29	270 (20.5)	54	23	16
1992–1996	1817 (21.9)	66	40	31	280 (21.2)	58	31	24
1997–2001	1505 (18.1)	69	43	35	271 (20.5)	68	37	28
2002–2006	1293 (15.6)	71	43	35	171 (13.0)	76	44	36
2007–2011	995 (12.0)	77	49	37	176 (13.3)	90	57	43
2012–2016	632 (7.6)	77	49	38	152 (11.5)	86	52	40
No surgery								
1987–1991	2435 (23.4)	18	8	7	407 (17.7)	20	7	5
1992–1996	1903 (18.3)	16	7	6	396 (17.2)	20	4	4
1997–2001	1630 (15.6)	16	5	4	386 (16.8)	20	4	2
2002–2006	1635 (15.7)	17	5	3	380 (16.5)	23	3	2
2007–2011	1557 (14.9)	20	6	4	436 (19.0)	26	5	3
2012–2016	1260 (12.1)	22	6	4	292 (12.7)	32	9	7

#### Surgical patients

The 5-year survival rate in patients who underwent surgical treatment for gastric non-cardia adenocarcinoma improved from 29% during 1987–1991 to 38% during 2012–2016 (Table 2, Fig. 2c). Similarly, increasing survival (31% to 44%) was observed for gastric cancers of any histology (Supplementary Table 2, Supplementary Fig. 2c).

#### Non-surgical patients

Among the patients without surgical treatment for gastric non-cardia adenocarcinoma, the 5-year survival declined from 7% during 1987–1991 to 4% during 2012–2016 (Table 2, Fig. 2e). The 5-year survival rate increased from 4% during 1987–1991 to 7% during 2012–2016 in those patients undergoing chemotherapy only, whereas the survival rate declined from 6% to 3% in those not going through any treatment (Supplementary Table 3, Supplementary Fig. 3c, 3e). In the analysis of all gastric histological gastric cancer types, the 5-year survival of patients not undergoing surgery increased from 11% during 1987–1991 to 15% during 2012–2016 (Supplementary Table 2, Supplementary Fig. 2e).

#### Cancer stage

The 5-year survival increased in gastric non-cardia adenocarcinoma patients from 45% to 57% in those with local cancer stage, from 17% to 26% in those with locally advanced cancer stage, and only slightly from 2% to 3% in those with advanced cancer stage between 1987–1991 and 2012–2016 (Table 3, Supplementary Fig. 4a, 4c, 4e).

**Table 3:** One-year-, 3-year-, and 5-year survival in histologically confirmed gastric adenocarcinoma (non-cardia and cardia) stratified by cancer stage during 1987–2016

	Gastric non-cardia adenocarcinoma				Gastric cardia adenocarcinoma			
	Patients	Survival in %			Patients	Survival in %		
Calendar period	Number (%)	1 year	3 years	5 years	Number (%)	1 year	3 years	5 years
Local								
1987–1991	1092 (30.3)	74	55	45	147 (24.5)	64	32	26
1992–1996	960 (26.6)	79	59	48	150 (25.0)	62	42	33
1997–2001	609 (16.9)	78	60	52	107 (17.9)	68	43	35
2002–2006	488 (13.5)	78	60	50	85 (14.2)	71	49	37
2007–2011	351 (9.7)	74	58	50	88 (14.7)	62	48	34
2012–2016	108 (3.0)	79	62	57	22 (3.7)	73	54	55
Locally advanced								
1987–1991	505 (22.7)	61	26	17	83 (17.7)	46	11	5
1992–1996	447 (20.1)	62	28	20	91 (19.4)	57	19	12
1997–2001	459 (20.6)	68	34	23	116 (24.7)	68	32	23
2002–2006	350 (15.7)	72	40	31	61 (13.0)	74	33	26
2007–2011	267 (12.0)	80	44	28	80 (17.1)	82	46	28
2012–2016	198 (8.9)	66	35	26	38 (8.1)	76	37	24
Advanced								
1987–1991	1892 (24.9)	16	3	2	290 (20.0)	14	2	1
1992–1996	1472 (19.3)	15	2	2	278 (19.1)	18	4	3
1997–2001	1203 (15.8)	16	4	3	258 (17.8)	18	3	1
2002–2006	1286 (16.9)	23	6	3	263 (18.1)	24	5	3
2007–2011	1243 (16.3)	30	12	7	273 (18.8)	34	8	6
2012–2016	514 (6.8)	18	4	3	90 (6.2)	20	6	4

### Survival trends in gastric cardia cancer

#### All patients

The 5- year survival rate in all patients with gastric cardia adenocarcinoma improved from 10% to 18% between the first and last calendar period (Table 2; Fig. 2b). In the analysis of all gastric cancers, the 5-year survival improved from 11% to 22% (Supplementary Table 2, Supplementary Fig. 2b).

#### Surgical patients

In patients who underwent surgical treatment for gastric cardia adenocarcinoma, the 5-year survival rate increased from 16% during 1987–1991 to 40% during 2012–2016 (Table 2; Fig. 2d). Similarly, increasing survival over time (18%–45%) was observed in all gastric cancers (Supplementary Table 2, Supplementary Fig. 2d).

#### Non-surgical patients

Among those who had not undergone surgical treatment for gastric cardia adenocarcinoma, the 5-year survival rate increased from 5% during 1987–1991 to 7% during 2012–2016 (Table 2; Fig. 2f). Similarly, an increase from 6% to 12% was observed in all gastric cancers (Supplementary Table 2, Supplementary Fig. 2f). Among non-surgical patients, the 5-year survival increased from 0% during 1987–1991 to 5% during 2012–2016 in those who underwent chemotherapy only, and from 5% to 9% between 1987–1991 and 2012–2016 in patients who received no treatment (Supplementary Table 3, Supplementary Fig. 3d, 3f).

#### Cancer stage

The 5-year survival increased in gastric cardia adenocarcinoma patients from 26% to 55% in those with local cancer stage, from 5% to 24% in those with locally advanced cancer stage, and from 1% to 4% in those with advanced cancer stage between 1987–1991 and 2012–2016 (Table 3, Supplementary Fig. 4b, 4d, 4f).

### Risk factors for 5-year mortality in gastric non-cardia cancer

In the multivariable cox-regression analysis of all patients with gastric non-cardia adenocarcinoma, earlier calendar periods (HR 1.08, 95% CI 1.02–1.15 in 1987–1991 vs 2012–2016), older age groups (HR 2.92, 95% CI 2.64–3.24, age 90 + vs age > 50), higher comorbidity score (HR 1.22, 95% CI 1.14–1.32, CCI 3 + vs 0), and male sex (HR 1.07, 95% CI 1.03–1.10, male vs female) were observed to be associated with an increased risk of mortality. The risk factors were similar in patients undergoing surgery and not undergoing surgery, with the exception that comorbidity was not a risk factor for mortality in those not undergoing surgery (Table 4). In the analysis of all gastric cancers, the risk factors were the same as in gastric adenocarcinoma, with comorbidity in addition in those not undergoing surgery (Supplementary Table 4).

**Table 4:** Multivariable cox-regression analysis of risk of mortality during 5-year follow-up in all histologically confirmed gastric non-cardia adenocarcinoma patients and stratified by surgical treatment

Covariate	Category	Gastric non- cardia adenocarcinoma	
		Number of patients (%)	HR (95% CI)
All patients			
Calendar period	1987–1991	4486 (24.0)	1.08 (1.02–1.15)
	1992–1996	3720 (19.9)	1.02 (0.96–1.08)
	1997–2001	3135 (16.8)	0.97 (0.91–1.03)
	2002–2006	2928 (15.6)	1.02 (0.95–1.08)
	2007–2011	2552 (13.6)	0.97 (0.91–1.04)
	2012–2016	1892 (10.1)	1 (Reference)
Age	> 50	1325 (7.1)	1 (Reference)
	50–59	2243 (12.0)	1.03 (0.95–1.12)
	60–69	4197 (22.4)	1.14 (1.06–1.22)
	70–79	5910 (31.6)	1.37 (1.28–1.47)
	80–89	4398 (23.5)	1.90 (1.77–2.04)
	90 +	640 (3.4)	2.92 (2.64–3.24)
Sex	Female	8864 (47.4)	1 (Reference)
	Male	9849 (52.6)	1.07 (1.03–1.10)
CCI	0	12,007 (64.2)	1 (Reference)
	1	4058 (21.7)	1.01 (0.97–1.05)
	2	1717 (9.2)	1.12 (1.06–1.18)
	3 +	931 (5.0)	1.22 (1.14–1.32)
Surgery			
Calendar period	1987–1991	2051 (24.7)	1.58 (1.41–1.77)
	1992–1996	1817 (21.9)	1.40 (1.24–1.56)
	1997–2001	1505 (18.1)	1.21 (1.07–1.36)
	2002–2006	1293 (15.6)	1.19 (1.06–1.35)
	2007–2011	995 (12.0)	1.03 (0.90–1.16)
	2012–2016	632 (7.6)	1 (Reference)
Age	> 50	728 (8.8)	1 (Reference)
	50–59	1186 (14.3)	1.01 (0.89–1.14)
	60–69	2129 (25.7))	1.11 (1.00–1.24)
	70–79	2777 (33.5)	1.37 (1.23–1.52)
	80–89	1410 (17.0)	1.83 (1.63–2.05)
	90 +	63 (0.8)	2.94 (2.22–3.88)
Sex	Female	3865 (46.6)	1 (Reference)
	Male	4428 (53.4)	1.10 (1.05–1.16)
CCI	0	5515 (66.5)	1 (Reference)
	1	1805 (21.8)	1.13 (1.06–1.21)
	2	653 (7.9)	1.25 (1.13–1.38)
	3 +	320 (3.9)	1.55 (1.36–1.77)
No surgery			
Calendar period	1987–1991	2435 (23.4)	1.05 (0.98–1.13)
	1992–1996	1903 (18.3)	1.13 (1.05–1.22)
	1997- 2001	1630 (15.6)	1.13 (1.05–1.22)
	2002–2006	1635 (15.7)	1.16 (1.08–1.25)
	2007–2011	1557 (14.9)	1.07 (0.99–1.15)
	2012–2016	1260 (12.1)	1 (Reference)
Age	> 50	597 (5.7)	1 (Reference)
	50–59	1057 (10.1)	1.03 (0.93–1.15)
	60–69	2068 (19.8)	1.13 (1.02 -1.24)
	70–79	3133 (30.1)	1.28 (1.17–1.40)
	80–89	2988 (28.7)	1.44 (1.33–1.58)
	90 +	577 (5.5)	1.65 (1.47–1.86)
Sex	Female	4999 (48.0)	1 (Reference)
	Male	5421 (52.0)	1.03 (0.99–1.08)
CCI	0	6492 (62.3)	1 (Reference)
	1	2253 (21.6)	0.95 (0.91–1.00)
	2	1064 (10.2)	0.99 (0.93–1.06)
	3 +	611 (5.9)	0.97 (0.89–1.05)

*CCI* Charlson Comorbidity Index

### Risk factors for 5-year mortality in gastric cardia cancer

In the multivariable cox-regression analysis of all patients with gastric cardia adenocarcinoma, the risk factors of mortality were earlier calendar periods (HR 1.49, 95% CI 1.30–1.71 in 1987–1991 vs 2012–2016), older age groups (HR 2.03, 95% CI 1.59–2.60, age 90 + vs > 50), male sex (HR 1.05, 95% CI 0.97–1.14, male vs female), and high comorbidity score (HR 1.33, 95% 1.14–1.55, CCI 3 + vs 0) (Table 5). The risk factors were similar in patients undergoing surgery and not undergoing surgery, with the exception that male sex was not a risk factor for mortality in those not undergoing surgery. The results were similar in all gastric cancers with the male sex being associated with poor survival in both non-surgical and surgical groups (Supplementary Table 5).

**Table 5:** Multivariable cox-regression analysis of risk of mortality during 5-year follow-up in all histologically confirmed gastric cardia adenocarcinoma patients and stratified by surgical treatment

Covariate	Category	Gastric cardia adenocarcinoma	
		Number of patients (%)	HR (95% CI)
All patients			
Calendar period	1987–1991	677 (18.7)	1.49 (1.30–1.71)
	1992–1996	676 (18.7)	1.36 (1.19–1.55)
	1997–2001	657 (18.2)	1.27 (1.11–1.45)
	2002–2006	551 (15.2)	1.24 (1.08–1.43)
	2007–2011	612 (16.9)	1.13 (0.98–1.29)
	2012–2016	444 (12.3)	1 (Reference)
Age	> 50	265 (7.3)	1 (Reference)
	50–59	523 (14.5)	0.91 (0.78–1.07)
	60–69	929 (25.7)	0.98 (0.84–1.14)
	70–79	1126 (31.1)	1.13 (0.97–1.30)
	80–89	678 (18.7)	1.59 (1.36–1.87)
	90 +	96 (2.7)	2.03 (1.59–2.60)
Sex	Female	1085 (30.0)	1 (Reference)
	Male	2532 (70.0)	1.05 (0.97–1.14)
CCI	0	2222 (61.4)	1 (Reference)
	1	817 (22.6)	1.07 (0.98–1.17)
	2	375 (10.4)	1.26 (1.12–1.42)
	3 +	203 (5.6)	1.33 (1.14–1.55)
Surgery			
Calendar period	1987–1991	270 (20.5)	2.22 (1.72–2.86)
	1992–1996	280 (21.2)	1.82 (1.42–2.35)
	1997–2001	271 (20.5)	1.48 (1.14–1.90)
	2002–2006	171 (13.0)	1.17 (0.88–1.54)
	2007–2011	176 (13.3)	0.86 (0.65–1.15)
	2012–2016	152 (11.5)	1 (Reference)
Age	> 50	117 (8.9)	1 (Reference)
	50–59	225 (17.0)	0.84 (0.63–1.10)
	60–69	409 (31.0)	1.00 (0.78–1.29)
	70–79	445 (33.7)	1.19 (0.92–1.52)
	80–89	118 (8.9)	1.45 (1.06–1.97)
	90 +	6 (0.5)	2.50 (1.09–5.77)
Sex	Female	361 (27.3)	1 (Reference)
	Male	959 (72.7)	1.22 (1.05–1.42)
CCI	0	905 (68.6)	1 (Reference)
	1	283 (21.4)	1.01 (0.85–1.19)
	2	94 (7.1)	1.13 (0.87–1.46)
	3 +	38 (2.9)	1.30 (0.85–1.85)
No surgery			
Calendar period	1987–1991	407 (17.7)	1.34 (1.14–1.57)
	1992–1996	396 (17.2)	1.36 (1.16–1.59)
	1997–2001	386 (16.8)	1.42 (1.21–1.66)
	2002–2006	380 (16.5)	1.28 (1.09–1.50)
	2007–2011	436 (19.0)	1.20 (1.03–1.40)
	2012–2016	292 (12.7)	1 (Reference)
Age	> 50	148 (6.4)	1 (Reference)
	50–59	298 (13.0)	0.90 (0.73–1.10)
	60–69	520 (22.6)	0.96 (0.80–1.16)
	70–79	681 (29.6)	1.02 (0.85–1.23)
	80–89	560 (24.4)	1.12 (0.93–1.36)
	90 +	90 (3.9)	1.24 (0.95–1.64)
Sex	Female	724 (31.5)	1 (Reference)
	Male	1573 (68.5)	0.98 (0.90–1.08)
CCI	0	1317 (57.3)	1 (Reference)
	1	534 (23.2)	1.07 (0.96–1.19)
	2	281 (12.2)	1.14 (1.00–1.31)
	3 +	165 (7.2)	1.11 (0.94–1.32)

*CCI* Charlson Comorbidity Index

## Discussion

This study indicates a declining survival in histologically confirmed gastric non-cardia adenocarcinoma but an improved overall survival for gastric cardia cancer during the last 3 decades in Finland. Significant improvement in 5-year survival was observed particularly among surgically treated patients as well as patients undergoing chemotherapy. Declining survival was seen among those non-cardia adenocarcinoma patients who did not receive any treatment. Earlier calendar period, older age, male sex, and higher comorbidity score were associated with poor prognosis in both gastric non-cardia and cardia cancers.

The main strength of this study is its population-based design, which reduced selection bias. The large cohort size allowed robust and reliable estimates, and the 100% complete follow-up of all patients based on national vital registration ensures that no misclassification occurred for the outcome variables. However, missing histology records for some patients led to their exclusion from the analysis of confirmed adenocarcinoma cases, and therefore, some selection bias is present in analyses of patients with adenocarcinoma. Another strength includes the highly accurate data retrieved from two independent and complete nationwide registries (FCR and HILMO) [19], which were combined using the immutable Finnish personal identity numbers assigned to all citizens, ensuring that all cases are captured. There were some missing values for oncological treatment status and tumor stage, and therefore, the estimates concerning chemotherapy patients and stage-specific analyses should be interpreted cautiously. Weaknesses include lack of TNM stage and more granular histological data, such as Laurén or WHO classification concerning adenocarcinoma patients.

The survival patterns of gastric adenocarcinoma prognosis in several European countries have been assessed previously. A Dutch study on gastric adenocarcinomas showed that 5-year survival for gastric non-cardia adenocarcinoma decreased from 22% to 14% [20]. A Swedish study suggested that the 5-year prognosis of gastric non-cardia adenocarcinoma was stable at 18% between 1990–1994 and 2012–2014 [13]. In the present study, the 5-year survival for confirmed gastric non-cardia adenocarcinomas decreased from 17% to 16% between 1987–1991 and 2012–2016. However, significant improvements in 5-year survival were observed in patients undergoing surgery or chemotherapy, as well as those with local or locally advanced disease, while those not receiving any treatment or advanced disease had worsening prognosis over time. Some of the improvements in prognosis can be explained by increased patient awareness and developments in surgical techniques [21]. Centralization of surgery might have improved treatment results [22], but gastric cancer surgery was formally centralized in Finland after the study period. As the proportion of surgically treated patients has declined over time, it is questionable whether surgical selection of patients has become even too strict. More accurate diagnostic imaging leads to better preoperative staging, and therefore, metastatic diseases are not operated on so often. It could be speculated that the worsening prognosis in no treatment group is due to the selection of the heathier patients for palliative chemotherapy. However, it remains unclear why the prognosis in no surgery group declined over time.

The recent trends of cardia adenocarcinoma prognosis have been previously studied in the Netherlands (stable at 10% from 1990 to 2005) [20] and Sweden (increased from 12% in 1990–1994 to 18% in 2012–2014) [13]. The present study suggests improved prognosis for cardia adenocarcinoma from 10% to 18% between 1987–1991 and 2012–2016, which is uplifting and in line with the Swedish mortality figures. Similar to non-cardia adenocarcinomas, their treatment has been centralized and neoadjuvant therapies have become more commonplace, improved prognosis in those undergoing surgery and oncological treatment. Opposed to non-cardia adenocarcinomas, the prognosis of those with advanced disease and receiving no treatment has also improved over time. While there are no clear explanations for this discrepancy, these findings may indicate differences in presenting symptoms or differences in cancer biology between non-cardia and cardia adenocarcinomas.

In a larger context, the prognosis of non-cardia gastric cancer of any histology has improved from 19% in 1987–1991 to 24% in 2012–2016, and from 11% in 1987–1991 to 24% in 2012–2016 for gastric cardia cancer. This is in line with registry studies from the United States (improvement from 26% to 29% during 2000s) [23], Europe (23% in 1999 to 25% in 2007) [10], and South Korea (43% in 1993–1995 to 74% in 2010–2014) [24]. While this overall improving survival in both gastric non-cardia and cardia is encouraging, these numbers include all histology of gastric cancer, such as neuroendocrine and gastrointestinal stromal tumors, which have a much better prognosis than adenocarcinomas [25, 26]. Increasing use of abdominal computed tomography might also have led to the more frequent discovery of gastric incidentalomas, which often include asymptomatic non-adenocarcinoma tumors with better prognosis and some of these might only be followed up clinically [27]. Therefore, some of the global improvement in gastric cancer survival might not completely reflect improved survival of gastric adenocarcinomas, but might also be related to increased detection of non-adenocarcinoma cancers of other histological types, thereby, increasing incidence and improving prognosis [4].

In addition to earlier calendar periods, older age groups, comorbidity, and male sex were observed to be risk factors for poor prognosis of both gastric non-cardia and cardia cancers. While the other factors are largely in line with the previous studies, the finding on the association of sex difference in poor prognosis of gastric cancer was rather unexpected. In the subgroup analyses, sex was associated with prognosis in only surgical gastric non-cardia and cardia adenocarcinoma. In Sweden, which is a relatively similar population compared to Finland, there was no difference in prognosis between male and female gastric non-cardia adenocarcinoma patients [13]. The higher incidence of gastric cancer in men results from genetic, environmental, and behavioral factors, including alcohol and tobacco consumption, differences in healthcare-seeking patterns, and utilization of health resources between men and women. Prevalence of *H. pylori* is, however, the most detrimental risk factor associated with poor prognosis in both sexes [28–31]. Estrogen in women might protect against H. pylori infection resulting in a lower incidence of gastric cancer in women [32–34]. These factors might explain the disparity observed in survival between the two sexes.

This study has several research and clinical implications. First, the declining prognosis in gastric non-cardia adenocarcinomas highlights the need for actions, such as further centralization of gastric cancer care, and evaluation and research of treatment options such as immunotherapies or cytoreductive surgery to further improve the prognosis of the disseminated disease. Second, the discrepancy in survival trend patterns between non-cardia and cardia cancers need further studies to understand the reasons these two cancer types are behaving differently. Finally, the differences in prognostic trends between gastric adenocarcinomas and all gastric cancers may indicate that optimistic estimates of survival trends may be obtained by studying gastric cancers without taking into account cancer histology.

In conclusion, this population-based, nationwide study on survival trends of gastric cancer in Finland indicates that the 5-year survival in patients with gastric non-cardia adenocarcinoma is declining but improving for cardia adenocarcinomas. However, the overall survival in all gastric cancers has improved during the last 3 decades in Finland. Earlier calendar period, older age, male sex, and higher comorbidity scores were poor prognostic factors for both gastric non-cardia and cardia cancer.

## Supplementary Information

Below is the link to the electronic supplementary material.

Supplementary file1 (DOCX 2123 KB)

## Data Availability

All presented data are available from THL/Findata, Finland. Data access to collaborators can be granted given that relevant government and health officials approve the collaborative study.
