# Supplementary material for: Survival trends in gastric cancer patients between 1987 and 2016: a population-based cohort study in Finland
Source: Gastric Cancer. 2022 Aug 7;25(6):989–1001. doi: 10.1007/s10120-022-01326-5 (PMC9587955; doi:10.1007/s10120-022-01326-5)
Supplement: Supplementary file 1 — Supplementary file1 (DOCX 2123 KB) [file 10120_2022_1326_MOESM1_ESM.docx]

**Supplementary Table 1. Gastric non-cardia and cardia cancer patients stratified by calendar period, age, sex, CCI, surgical treatment, chemotherapy and cancer stage.**

| **Variable** | **Gastric non-cardia cancer n (%)** | **Gastric cardia cancer n (%)** | **Total n (%)** |
| --- | --- | --- | --- |
| **Total** | 22502 (83.6) | 4418 (16.4) | 26920 (100) |
| **Calendar period** | | | |
| 1987-1991 | 5145 (22.9) | 740 (16.7) | 5885 (21.9) |
| 1992-1996 | 4317 (19.2) | 727 (16.5) | 5044 (18.7) |
| 1997- 2001 | 3682 (16.4) | 778 (17.6) | 4460 (16.6) |
| 2002-2006 | 3493 (15.5) | 704 (15.9) | 4197 (15.6) |
| 2007- 2011 | 3169 (14.1) | 763 (17.3) | 3932 (14.6) |
| 2012- 2016 | 2696 (12.0) | 706 (16.0) | 3402 (12.6) |
| **Age** | | | |
| >50 | 1644 (7.3) | 312 (7.1) | 1956 (7.3) |
| 50-59 | 2789 (12.4) | 636 (14.4) | 3425 (12.7) |
| 60-69 | 5181 (23.0) | 1147 (26.0) | 6328 (23.5) |
| 70-79 | 7067 (31.4) | 1382 (31.3) | 8449 (31.4) |
| 80-89 | 5099 (22.7) | 831 (18.8) | 5930 (22.0) |
| 90+ | 722 (3.2) | 110 (2.5) | 832 (3.1) |
| **Sex** | | | |
| Female | 10800 (48.0) | 1421 (32.2) | 12221 (45.4) |
| Male | 11702 (52.0) | 2997 (67.8) | 14699 (54.6) |
| **CCI** | | | |
| 0 | 13967 (62.1) | 2554 (57.8) | 16521 (61.4) |
| 1 | 5102 (22.7) | 1074 (24.3) | 6176 (22.9) |
| 2 | 2207 (9.8) | 493 (11.2) | 2700 (10.0) |
| 3+ | 1226 (5.4) | 297 (6.7) | 1523 (5.7) |
| **Surgery** | | | |
| Yes | 9069 (40.3) | 1478 (33.5) | 10547 (39.2) |
| No | 13433 (59.7) | 2940 (66.5) | 16373 (60.8) |
| **Chemotherapy** | | | |
| No | 13589 (60.4) | 2311 (52.3) | 15900 (59.1) |
| Yes | 2881 (12.8) | 714 (16.2) | 3595 (13.4) |
| Missing | 6032 (26.8) | 1393 (31.5) | 7425 (27.6) |
| **Stage** | | | |
| Local | 4091 (18.2) | 618 (14.0) | 4709 (17.5) |
| Locally advanced | 2269 (10.1) | 475 (10.8) | 2744 (10.2) |
| Advanced | 7831 (34.8) | 1503 (34.0) | 9334 (34.7) |
| Unclear or missing | 8311 (36.9) | 1822 (41.2) | 10133 (37.6) |

**CCI:** Charlson Comorbidity Index

**Supplementary Table 2. One- year, 3-years and 5- years survival in gastric cancer (cardia and non-cardia) stratified by treatment during 1987-2016.**

|  | **Gastric non- cardia cancer** | | | | **Gastric cardia cancer** | | | |
| --- | --- | --- | --- | --- | --- | --- | --- | --- |
|  | **Patients** | **Survival in %** | | | **Patients** | **Survival in %** | | |
| **Calendar period** | **Number (%)** | **1 year** | **3 years** | **5 years** | **Number (%)** | **1 year** | **3 years** | **5 years** |
| **All patients** | | | | | | | | |
| 1987-1991 | 5145 (22.9) | 41 | 25 | 19 | 740 (16.7) | 36 | 16 | 11 |
| 1992-1996 | 4317 (19.2) | 43 | 26 | 21 | 727 (16.5) | 36 | 17 | 13 |
| 1997- 2001 | 3682 (16.4) | 45 | 27 | 23 | 778 (17.6) | 42 | 22 | 16 |
| 2002-2006 | 3493 (15.5) | 46 | 27 | 22 | 704 (15.9) | 43 | 23 | 18 |
| 2007- 2011 | 3169 (14.1) | 46 | 27 | 23 | 763 (17.3) | 48 | 25 | 19 |
| 2012- 2016 | 2696 (12.0) | 47 | 28 | 24 | 706 (16.0) | 51 | 26 | 22 |
| **Surgery** | | | | | | | | |
| 1987-1991 | 2216 (24.4) | 63 | 39 | 31 | 283 (19.1) | 55 | 25 | 18 |
| 1992-1996 | 1950 (21.5) | 67 | 41 | 33 | 293 (19.8) | 56 | 31 | 24 |
| 1997- 2001 | 1627 (17.9) | 70 | 45 | 37 | 294 (19.9) | 68 | 40 | 31 |
| 2002-2006 | 1395 (15.4) | 72 | 46 | 37 | 206 (13.9) | 78 | 50 | 42 |
| 2007- 2011 | 1085 (12.0) | 78 | 51 | 40 | 196 (13.3) | 87 | 56 | 45 |
| 2012- 2016 | 796 (8.8) | 79 | 53 | 44 | 206 (13.9) | 89 | 59 | 45 |
| **No surgery** | | | | | | | | |
| 1987-1991 | 2929 (21.8) | 24 | 14 | 11 | 457 (15.5) | 23 | 8 | 6 |
| 1992-1996 | 2367 (17.6) | 24 | 14 | 11 | 434 (14.8) | 24 | 10 | 6 |
| 1997- 2001 | 2055 (15.3) | 25 | 14 | 12 | 484 (16.5) | 26 | 11 | 7 |
| 2002-2006 | 2098 (15.6) | 27 | 15 | 13 | 498 (16.9) | 29 | 11 | 8 |
| 2007- 2011 | 2084 (15.5) | 31 | 17 | 15 | 567 (19.3) | 34 | 13 | 10 |
| 2012- 2016 | 1900 (14.1) | 33 | 18 | 15 | 500 (17.0) | 36 | 14 | 12 |

**Supplementary Table 3. One-year, 3-year-, and 5-year survival in histologically confirmed gastric adenocarcinoma (non-cardia and cardia) stratified by treatment type during 1987-2016.**

|  | **Gastric non- cardia adenocarcinoma** | | | | **Gastric cardia adenocarcinoma** | | | |
| --- | --- | --- | --- | --- | --- | --- | --- | --- |
|  | **Patients** | **Survival in %** | | | **Patients** | **Survival in %** | | |
| **Calendar period** | **Number (%)** | **1 year** | **3 years** | **5 years** | **Number (%)** | **1 year** | **3 years** | **5 years** |
| **Surgery** | | | | | | | | |
| 1987-1991 | 2051 (24.7) | 62 | 37 | 29 | 270 (20.5) | 54 | 23 | 16 |
| 1992-1996 | 1817 (21.9) | 66 | 40 | 31 | 280 (21.2) | 56 | 32 | 24 |
| 1997- 2001 | 1505 (18.1) | 69 | 43 | 35 | 271 (20.5) | 68 | 38 | 28 |
| 2002-2006 | 1293 (15.6) | 71 | 44 | 35 | 171 (13.0) | 76 | 44 | 36 |
| 2007- 2011 | 995 (12.0) | 77 | 50 | 37 | 176 (13.3) | 86 | 58 | 43 |
| 2012- 2016 | 632 (7.6) | 77 | 50 | 38 | 152 (11.5) | 90 | 54 | 40 |
| **Chemotherapy only** | | | | | | | | |
| 1987-1991 | 125 (9.1) | 28 | 6 | 4 | 25 (6.3) | 16 | 0 | 0 |
| 1992-1996 | 122 (8.9) | 32 | 4 | 1 | 31 (7.8) | 32 | 0 | 0 |
| 1997- 2001 | 238 (17.3) | 26 | 4 | 3 | 66 (16.5) | 34 | 4 | 2 |
| 2002-2006 | 302 (22.0) | 34 | 6 | 2 | 103 (25.8) | 30 | 2 | 2 |
| 2007- 2011 | 357 (26.0) | 38 | 9 | 6 | 115 (28.8) | 48 | 6 | 3 |
| 2012- 2016 | 229 (16.7) | 38 | 10 | 7 | 59 (14.8) | 50 | 8 | 5 |
| **No treatment** | | | | | | | | |
| 1987-1991 | 2015 (29.5) | 16 | 8 | 6 | 332 (23.7) | 20 | 6 | 5 |
| 1992-1996 | 1537 (22.5) | 14 | 7 | 5 | 319 (22.7) | 18 | 4 | 3 |
| 1997- 2001 | 1061 (15.5) | 13 | 5 | 4 | 245 (17.5) | 16 | 4 | 2 |
| 2002-2006 | 1013 (14.8) | 13 | 4 | 3 | 215 (15.3) | 18 | 3 | 1 |
| 2007- 2011 | 859 (12.6) | 13 | 4 | 3 | 225 (16.0) | 16 | 4 | 2 |
| 2012- 2016 | 339 (5.0) | 12 | 3 | 3 | 67 (4.8) | 24 | 9 | 9 |

**Supplementary Table 4. Multivariable cox-regression analysis of risk of mortality during 5-year follow-up in all gastric non- cardia cancer patients and stratified by surgical treatment.**

| **Covariate** | **Category** | **Gastric non- cardia cancer** | |
| --- | --- | --- | --- |
|  | | **Number of patients (%)** | **HR (95% CI)** |
| **All patients** | | | |
| **Calendar period** | 1987-1991 | 5145 (22.9) | 1.24 (1.17-1.30) |
|  | 1992-1996 | 4317 (19.2) | 1.16 (1.09-1.22) |
|  | 1997- 2001 | 3682 (16.4) | 1.07 (1.01-1.13) |
|  | 2002-2006 | 3493 (15.5) | 1.07 (1.01-1.14) |
|  | 2007- 2011 | 3169 (14.1) | 1.02 (0.96-1.08) |
|  | 2012- 2016 | 2696 (12.0) | 1 (Reference) |
| **Age** | >50 | 1644 (7.3) | 1 (Reference) |
|  | 50-59 | 2789 (12.4) | 1.08 (0.99-1.16) |
|  | 60-69 | 5181 (23.0) | 1.23 (1.14-1.31) |
|  | 70-79 | 7067 (31.4) | 1.49 (1.40-1.59) |
|  | 80-89 | 5099 (22.7) | 2.15 (2.01-2.30) |
|  | 90+ | 722 (3.2) | 3.30 (3.00-3.64) |
| **Sex** | Female | 10800 (48.0) | 1 (Reference) |
|  | Male | 11702 (52.0) | 1.13 (1.10-1.17) |
| **CCI** | 0 | 13967 (62.1) | 1 (Reference) |
|  | 1 | 5102 (22.7) | 1.08 (1.04-1.12) |
|  | 2 | 2207 (9.8) | 1.16 (1.11-1.22) |
|  | 3+ | 1226 (5.4) | 1.26 (1.18-1.35) |
| **Surgery** | | | |
| **Calendar period** | 1987-1991 | 2216 (24.4) | 1.72 (1.54-1.92) |
|  | 1992-1996 | 1950 (21.5) | 1.54 (1.38-1.71) |
|  | 1997- 2001 | 1627 (17.9) | 1.31 (1.17-1.47) |
|  | 2002-2006 | 1395 (15.4) | 1.27 (1.14-1.43) |
|  | 2007- 2011 | 1085 (12.0) | 1.09 (0.96-1.22) |
|  | 2012- 2016 | 796 (8.8) | 1 (Reference) |
| **Age** | >50 | 793 (8.7) | 1 (Reference) |
|  | 50-59 | 1314 (14.5) | 0.99 (0.88-1.12) |
|  | 60-69 | 2365 (26.1) | 1.13 (1.02-1.26) |
|  | 70-79 | 3013 (33.2) | 1.41 (1.27-1.57) |
|  | 80-89 | 1516 (16.7) | 1.93 (1.72-2.15) |
|  | 90+ | 68 (0.7) | 3.09 (2.36-4.04) |
| **Sex** | Female | 4262 (47.0) | 1 (Reference) |
|  | Male | 4807 (53.0) | 1.12 (1.06-1.18) |
| **CCI** | 0 | 5995 (66.1) | 1 (Reference) |
|  | 1 | 1992 (22.0) | 1.16 (1.09-1.24) |
|  | 2 | 723 (8.0) | 1.27 (1.16-1.40) |
|  | 3+ | 359 (4.0) | 1.55 (1.36-1.76) |
| **No surgery** | | | |
| **Calendar period** | 1987-1991 | 2929 (21.8) | 1.24 (1.16-1.32) |
|  | 1992-1996 | 2367 (17.6) | 1.24 (1.16-1.32) |
|  | 1997- 2001 | 2055 (15.3) | 1.19 (1.12-1.28) |
|  | 2002-2006 | 2098 (15.6) | 1.17 (1.09-1.25) |
|  | 2007- 2011 | 2084 (15.5) | 1.07 (1.00-1.14) |
|  | 2012- 2016 | 1900 (14.1) | 1 (Reference) |
| **Age** | >50 | 851 (6.3) | 1 (Reference) |
|  | 50-59 | 1475 (11.0) | 1.14 (1.03-1.26) |
|  | 60-69 | 2816 (21.0) | 1.31 (1.20-1.44) |
|  | 70-79 | 4054 (30.2) | 1.56 (1.43-1.70) |
|  | 80-89 | 3583 (26.7) | 2.00 (1.83-2.18) |
|  | 90+ | 654 (4.9) | 2.44 (2.18-2.73) |
| **Sex** | Female | 6538 (48.7) | 1 (Reference) |
|  | Male | 6895 (51.3) | 1.16 (1.12-1.21) |
| **CCI** | 0 | 7972 (59.3) | 1 (Reference) |
|  | 1 | 3110 (23.2) | 1.01 (0.96-1.05) |
|  | 2 | 1484 (11.0) | 1.02 (0.96-1.09) |
|  | 3+ | 867 (6.5) | 1.02 (0.95-1.10) |

**CCI:** Charlson Comorbidity Index

**Supplementary Table 5. Multivariable cox-regression analysis of risk of mortality during 5-year follow-up in all gastric cardia cancer patients and stratified by surgical treatment.**

| **Covariate** | **Category** | **Gastric cardia cancer** | |
| --- | --- | --- | --- |
|  | | **Number of patients (%)** | **HR (95% CI)** |
| **All patients** | | | |
| **Calendar period** | 1987-1991 | 740 (16.7) | 1.48 (1.31-1.66) |
|  | 1992-1996 | 727 (16.5) | 1.39 (1.24-1.57) |
|  | 1997- 2001 | 778 (17.6) | 1.23 (1.10-1.38) |
|  | 2002-2006 | 704 (15.9) | 1.12 (0.99-1.26) |
|  | 2007- 2011 | 763 (17.3) | 1.04 (0.93-1.17) |
|  | 2012- 2016 | 706 (16.0) | 1 (Reference) |
| **Age** | >50 | 312 (7.1) | 1 (Reference) |
|  | 50-59 | 636 (14.4) | 0.94 (0.80-1.09) |
|  | 60-69 | 1147 (26.0) | 1.02 (0.88-1.18) |
|  | 70-79 | 1382 (31.3) | 1.19 (1.03-1.37) |
|  | 80-89 | 831 (18.8) | 1.72 (1.49-2.00) |
|  | 90+ | 110 (2.5) | 2.46 (1.95-3.10) |
| **Sex** | Female | 1421 (32.2) | 1 (Reference) |
|  | Male | 2997 (67.8) | 1.17 (1.09-1.26) |
| **CCI** | 0 | 2554 (57.8) | 1 (Reference) |
|  | 1 | 1074 (24.3) | 1.05 (0.97-1.14) |
|  | 2 | 493 (11.2) | 1.18 (1.06-1.31) |
|  | 3+ | 297 (6.7) | 1.31 (1.14-1.49) |
| **Surgery** | | | |
| **Calendar period** | 1987-1991 | 283 (19.1) | 2.32 (1.83-2.93) |
|  | 1992-1996 | 293 (19.8) | 2.06 (1.63-2.60) |
|  | 1997- 2001 | 294 (19.9) | 1.57 (1.24-1.98) |
|  | 2002-2006 | 206 (13.9) | 1.11 (0.85-1.44) |
|  | 2007- 2011 | 196 (13.3) | 0.90 (0.69-1.18) |
|  | 2012- 2016 | 206 (13.9) | 1 (Reference) |
| **Age** | >50 | 124 (8.4) | 1 (Reference) |
|  | 50-59 | 243 (16.4) | 0.85 (0.64-1.11) |
|  | 60-69 | 469 (31.7) | 0.99 (0.77-1.27) |
|  | 70-79 | 499 (33.8) | 1.18 (0.92-1.51) |
|  | 80-89 | 136 (9.2) | 1.48 (1.10-2.00) |
|  | 90+ | 7 (0.5) | 3.05 (1.40-6.63) |
| **Sex** | Female | 427 (28.9) | 1 (Reference) |
|  | Male | 1051 (71.1) | 1.29 (1.12-1.49) |
| **CCI** | 0 | 987 (66.8) | 1 (Reference) |
|  | 1 | 334 (22.6) | 1.00 (0.85-1.18) |
|  | 2 | 111 (7.5) | 1.06 (0.83-1.36) |
|  | 3+ | 46 (3.1) | 1.46 (1.03-2.08) |
| **No surgery** | | | |
| **Calendar period** | 1987-1991 | 457 (15.5) | 1.33 (1.16-1.53) |
|  | 1992-1996 | 434 (14.8) | 1.34 (1.17-1.54) |
|  | 1997- 2001 | 484 (16.5) | 1.27 (1.11-1.45) |
|  | 2002-2006 | 498 (16.9) | 1.12 (0.98-1.28) |
|  | 2007- 2011 | 567 (19.3) | 1.05 (0.92-1.19) |
|  | 2012- 2016 | 500 (17.0) | 1 (Reference) |
| **Age** | >50 | 188 (6.4) | 1 (Reference) |
|  | 50-59 | 393 (13.4) | 0.95 (0.79-1.15) |
|  | 60-69 | 678 (23.1) | 1.08 (0.91-1.28) |
|  | 70-79 | 883 (30.0) | 1.20 (1.01-1.42) |
|  | 80-89 | 695 (23.6) | 1.46 (1.23-1.74) |
|  | 90+ | 103 (3.5) | 1.83 (1.42-2.36) |
| **Sex** | Female | 994 (33.8) | 1 (Reference) |
|  | Male | 1946 (66.2) | 1.20 (1.10-1.30) |
| **CCI** | 0 | 1567 (53.3) | 1 (Reference) |
|  | 1 | 740 (25.2) | 1.01 (0.92-1.11) |
|  | 2 | 382 (13.0) | 1.03 (0.92-1.17) |
|  | 3+ | 251 (8.5) | 1.02 (0.88-1.18) |

**CCI:** Charlson Comorbidity Index

**a. Gastric non-cardia cancer**

**b. Gastric cardia cancer**

**Supplementary Figure 1:** Graphs showing number of gastric non-cardia cancer (a) and gastric cardia cancer (b) patients diagnosed in Finland between 1987 to 2016. The curves show the number of patients diagnosed with cancer (blue line) and proportion of patients undergoing surgery (orange line).


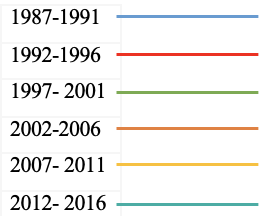

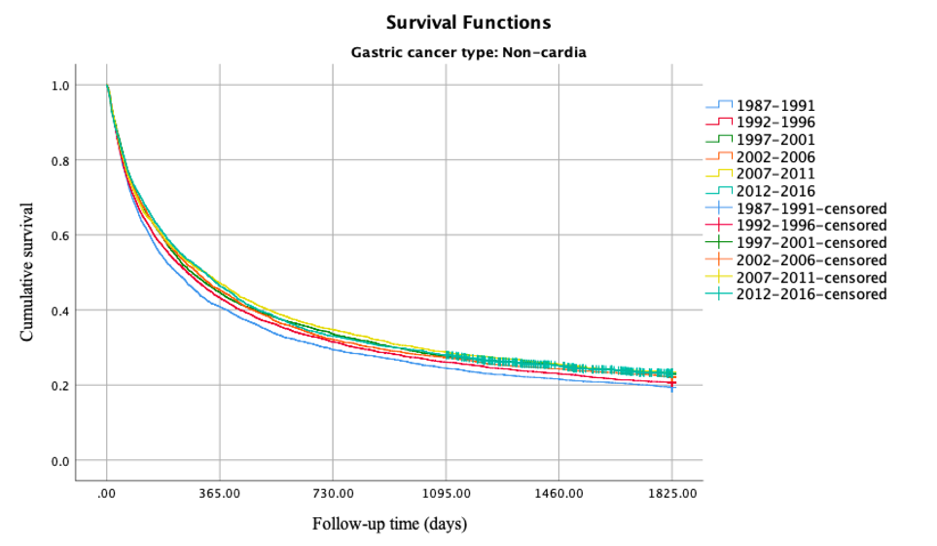


a. Non-cardia cancer, all patients

| 1987-1991 (n) | 5145 | 2100 | 1512 | 1260 | 1109 | 995 |
| --- | --- | --- | --- | --- | --- | --- |
| 1992-1996 (n) | 4317 | 1866 | 1359 | 1126 | 992 | 890 |
| 1997- 2001 (n) | 3682 | 1644 | 1236 | 1015 | 932 | 844 |
| 2002-2006 (n) | 3493 | 1582 | 1122 | 954 | 848 | 773 |
| 2007- 2011 (n) | 3169 | 1494 | 1100 | 913 | 808 | 733 |
| 2012- 2016 (n) | 2696 | 1250 | 890 | 755 | 591 | 402 |


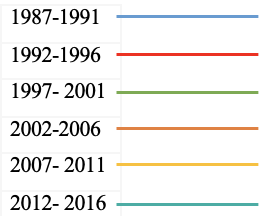

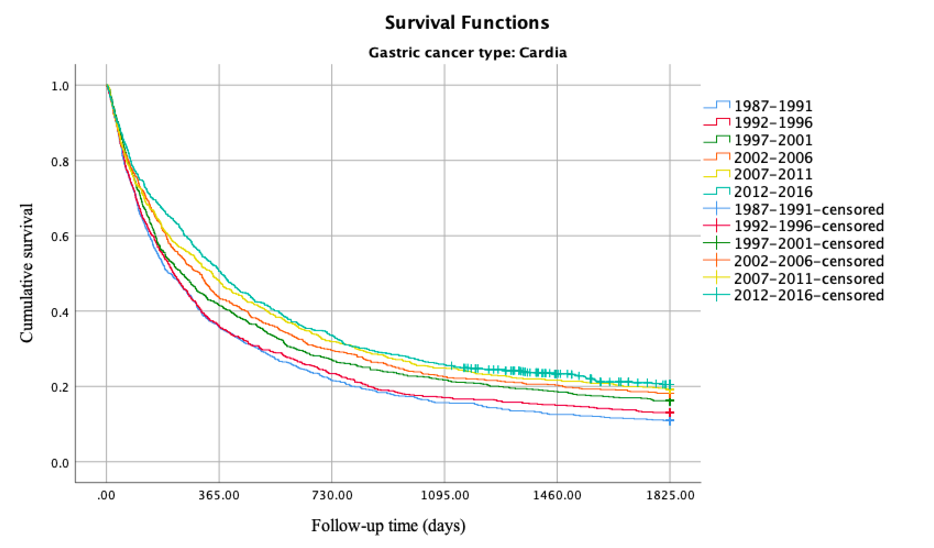


b. Cardia cancer, all patients

| 1987-1991 (n) | 740 | 264 | 160 | 116 | 93 | 80 |
| --- | --- | --- | --- | --- | --- | --- |
| 1992-1996 (n) | 727 | 262 | 170 | 124 | 109 | 94 |
| 1997- 2001 (n) | 778 | 323 | 210 | 169 | 145 | 125 |
| 2002-2006 (n) | 704 | 307 | 208 | 159 | 143 | 127 |
| 2007- 2011 (n) | 763 | 365 | 243 | 190 | 165 | 146 |
| 2012- 2016 (n) | 706 | 357 | 237 | 182 | 127 | 85 |


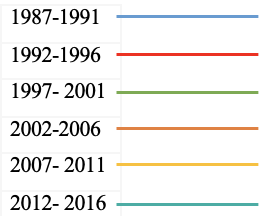

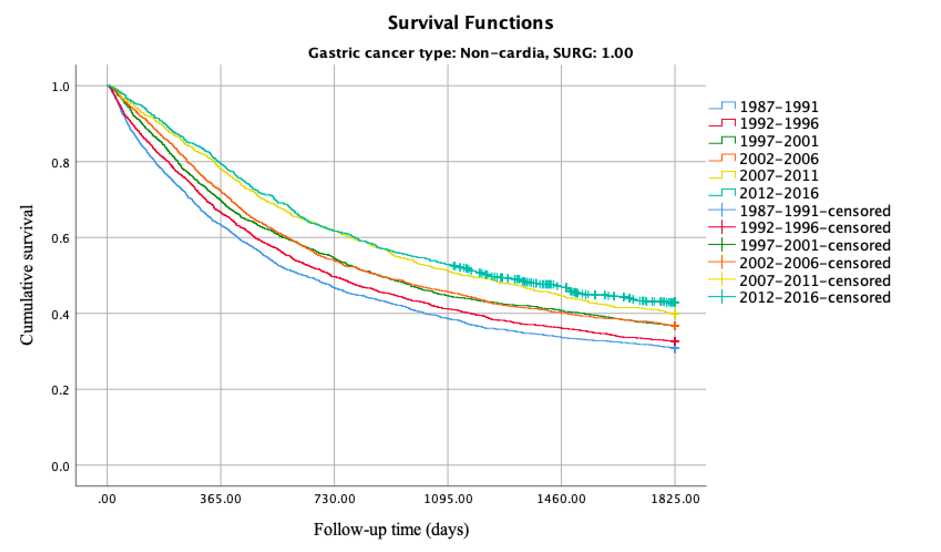


c. Non-cardia cancer, surgery

| 1987-1991 (n) | 2216 | 1403 | 1035 | 857 | 746 | 682 |
| --- | --- | --- | --- | --- | --- | --- |
| 1992-1996 (n) | 1950 | 1298 | 968 | 803 | 704 | 635 |
| 1997- 2001 (n) | 1627 | 1135 | 887 | 726 | 663 | 597 |
| 2002-2006 (n) | 1395 | 1008 | 753 | 638 | 560 | 511 |
| 2007- 2011 (n) | 1085 | 847 | 670 | 556 | 484 | 431 |
| 2012- 2016 (n) | 796 | 633 | 491 | 421 | 322 | 217 |


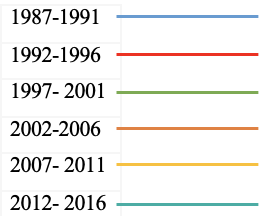

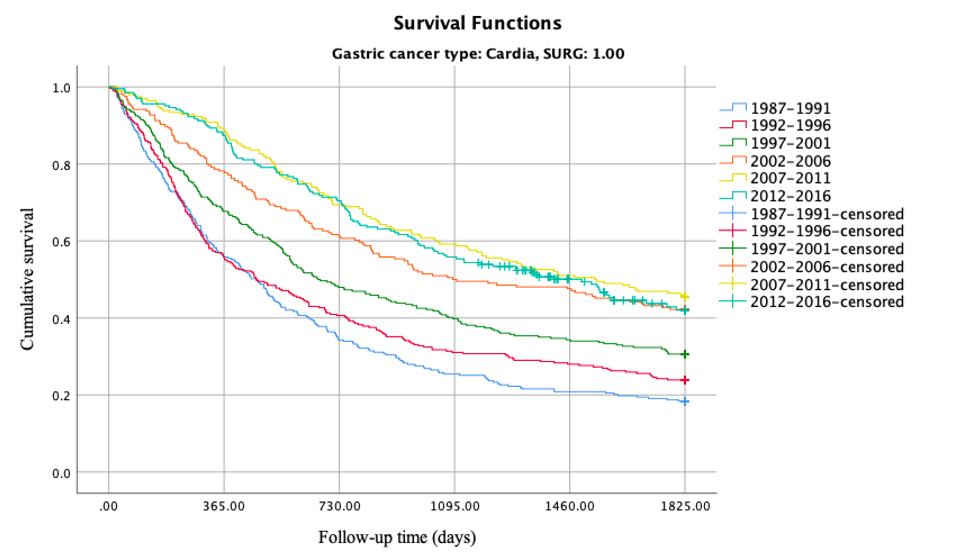


d. Cardia cancer, surgery

| 1987-1991 (n) | 283 | 158 | 97 | 72 | 59 | 51 |
| --- | --- | --- | --- | --- | --- | --- |
| 1992-1996 (n) | 293 | 164 | 119 | 91 | 82 | 69 |
| 1997- 2001 (n) | 294 | 199 | 141 | 117 | 101 | 89 |
| 2002-2006 (n) | 206 | 161 | 126 | 103 | 98 | 86 |
| 2007- 2011 (n) | 196 | 174 | 136 | 116 | 100 | 88 |
| 2012- 2016 (n) | 206 | 180 | 145 | 115 | 75 | 48 |


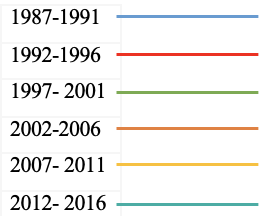

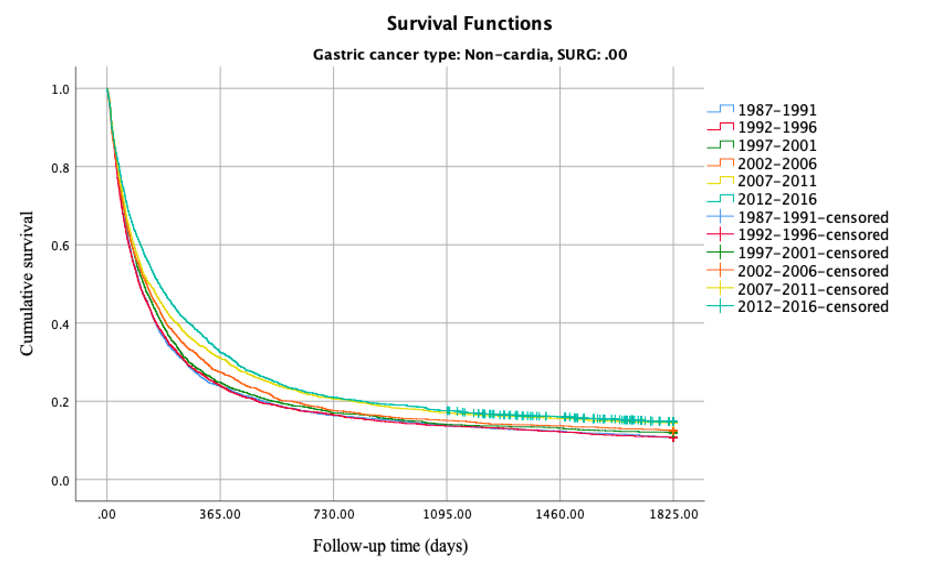


e. Non-cardia cancer, no surgery

| 1987-1991 (n) | 2929 | 698 | 477 | 403 | 363 | 312 |
| --- | --- | --- | --- | --- | --- | --- |
| 1992-1996 (n) | 2367 | 568 | 391 | 323 | 288 | 254 |
| 1997- 2001 (n) | 2055 | 509 | 349 | 289 | 269 | 246 |
| 2002-2006 (n) | 2098 | 574 | 369 | 316 | 288 | 261 |
| 2007- 2011 (n) | 2084 | 647 | 430 | 357 | 324 | 301 |
| 2012- 2016 (n) | 1900 | 617 | 399 | 334 | 269 | 184 |


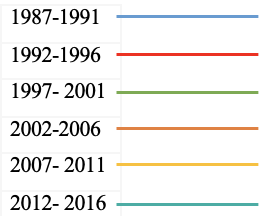

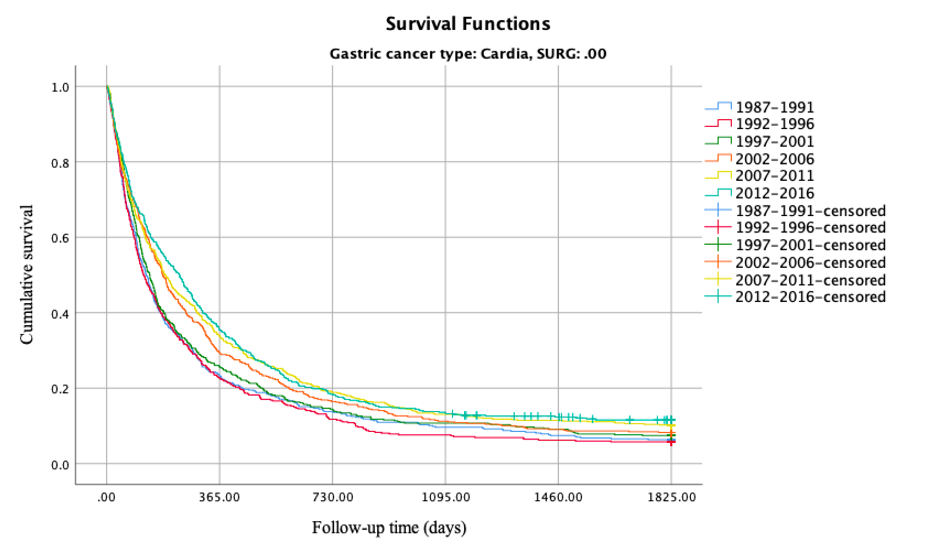


f. Cardia cancer, no surgery

| 1987-1991 (n) | 457 | 106 | 63 | 44 | 34 | 28 |
| --- | --- | --- | --- | --- | --- | --- |
| 1992-1996 (n) | 434 | 98 | 51 | 33 | 27 | 24 |
| 1997-2001 (n) | 484 | 124 | 69 | 52 | 44 | 35 |
| 2002-2006 (n) | 498 | 146 | 82 | 56 | 45 | 40 |
| 2007- 2011 (n) | 567 | 191 | 107 | 74 | 65 | 57 |
| 2012- 2016 (n) | 500 | 177 | 92 | 67 | 52 | 36 |

**Supplementary Figure 2:** Kaplan-Meier survival curves showing 5-year survival rate for gastric non-cardia cancer (a) and gastric cardia cancer (b) in total, for gastric non-cardia cancer (c) and gastric cardia cancer (d) undergoing surgery, and for gastric non-cardia cancer (e) and gastric cardia cancer (f) not undergoing surgery, stratified by 5-year time periods.


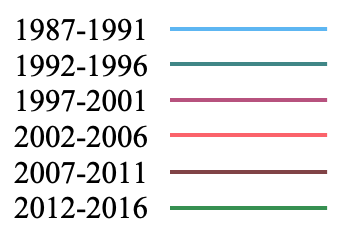

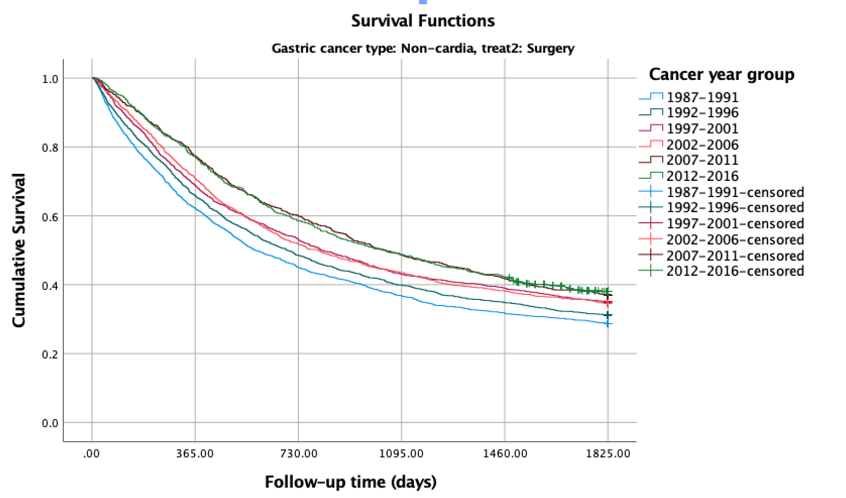


1. Non-cardia adenocarcinoma, surgery

| 1987-1991 (n) | 2051 | 1276 | 923 | 755 | 650 | 588 |
| --- | --- | --- | --- | --- | --- | --- |
| 1992-1996 (n) | 1817 | 1197 | 882 | 725 | 633 | 566 |
| 1997- 2001 (n) | 1505 | 1037 | 800 | 648 | 587 | 526 |
| 2002-2006 (n) | 1293 | 918 | 671 | 563 | 493 | 446 |
| 2007- 2011 (n) | 995 | 770 | 596 | 485 | 415 | 367 |
| 2012- 2016 (n) | 632 | 488 | 370 | 308 | 267 | 182 |


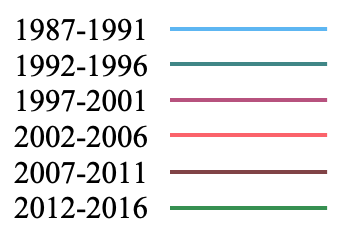

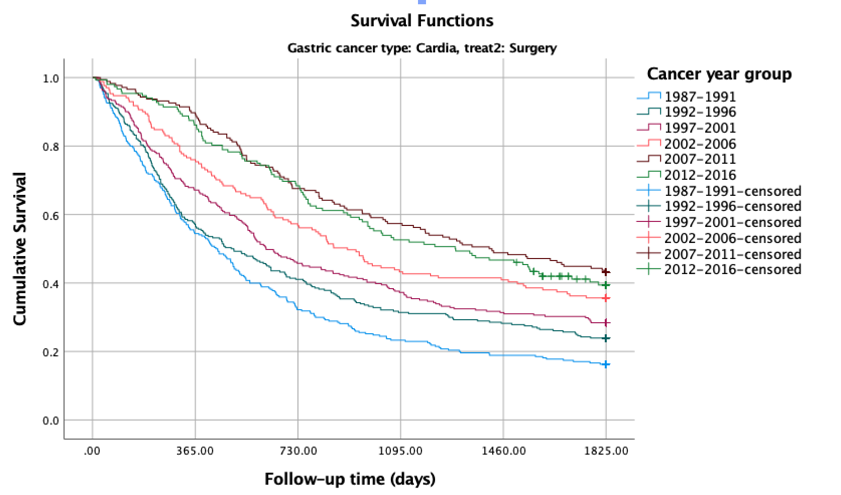


1. Cardia adenocarcinoma, surgery

| 1987-1991 (n) | 270 | 147 | 87 | 63 | 51 | 43 |
| --- | --- | --- | --- | --- | --- | --- |
| 1992-1996 (n) | 280 | 160 | 115 | 88 | 79 | 66 |
| 1997- 2001 (n) | 271 | 182 | 124 | 101 | 85 | 76 |
| 2002-2006 (n) | 171 | 130 | 97 | 74 | 70 | 60 |
| 2007- 2011 (n) | 176 | 157 | 119 | 101 | 86 | 75 |
| 2012- 2016 (n) | 152 | 131 | 104 | 80 | 71 | 45 |


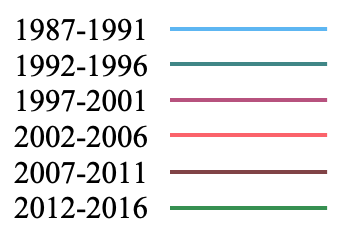
**
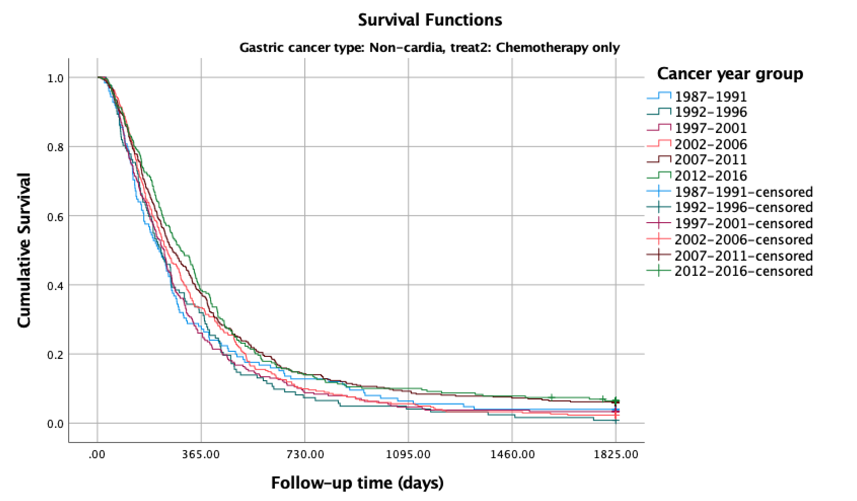
**

1. Non-cardia adenocarcinoma, chemotherapy only

| 1987-1991 (n) | 125 | 35 | 16 | 8 | 5 | 4 |
| --- | --- | --- | --- | --- | --- | --- |
| 1992-1996 (n) | 122 | 39 | 9 | 5 | 3 | 0 |
| 1997- 2001 (n) | 238 | 62 | 21 | 11 | 9 | 7 |
| 2002-2006 (n) | 302 | 101 | 30 | 17 | 10 | 6 |
| 2007- 2011 (n) | 357 | 134 | 51 | 33 | 26 | 20 |
| 2012- 2016 (n) | 229 | 88 | 32 | 23 | 18 | 12 |


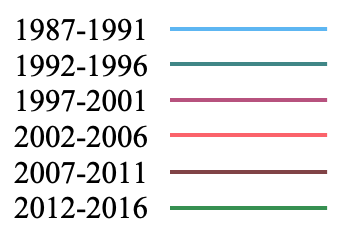
 **
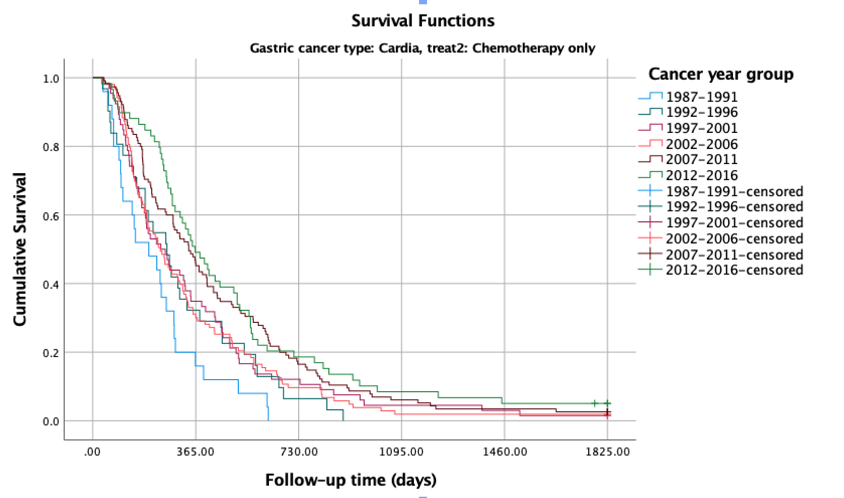
**

1. Cardia adenocarcinoma, Chemotherapy only

| 1987-1991 (n) | 25 | 4 | - | - | - | - |
| --- | --- | --- | --- | --- | --- | --- |
| 1992-1996 (n) | 31 | 10 | 2 | - | - | - |
| 1997- 2001 (n) | 66 | 23 | 8 | 3 | 2 | 0 |
| 2002-2006 (n) | 103 | 31 | 10 | 2 | 0 | 0 |
| 2007- 2011 (n) | 115 | 52 | 19 | 7 | 4 | 2 |
| 2012- 2016 (n) | 59 | 29 | 11 | 5 | 3 | 1 |


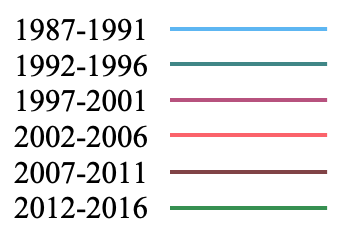
**
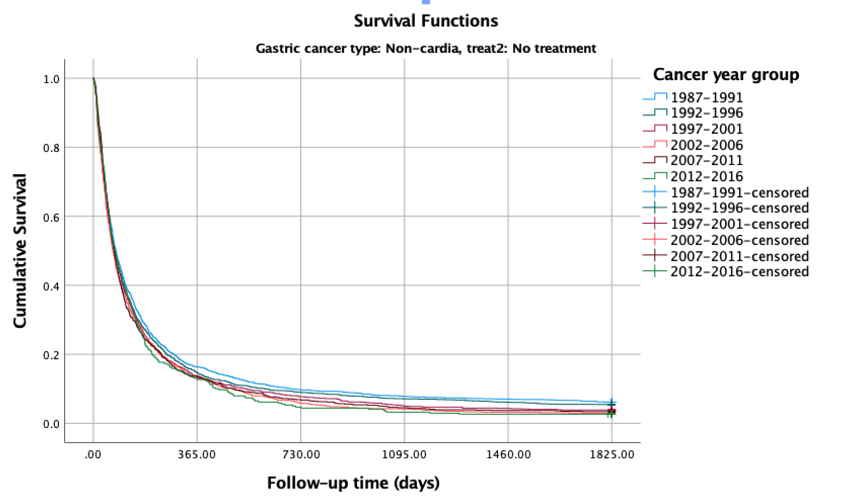
**

1. Non-cardia adenocarcinoma, no treatment

| 1987-1991 (n) | 2015 | 330 | 196 | 158 | 142 | 123 |
| --- | --- | --- | --- | --- | --- | --- |
| 1992-1996 (n) | 1537 | 227 | 139 | 109 | 94 | 81 |
| 1997- 2001 (n) | 1061 | 146 | 82 | 54 | 46 | 40 |
| 2002-2006 (n) | 1013 | 132 | 59 | 42 | 33 | 27 |
| 2007- 2011 (n) | 859 | 116 | 58 | 39 | 32 | 28 |
| 2012- 2016 (n) | 339 | 44 | 16 | 11 | 9 | 7 |


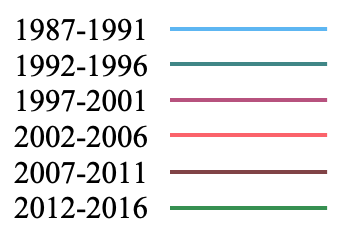
 **
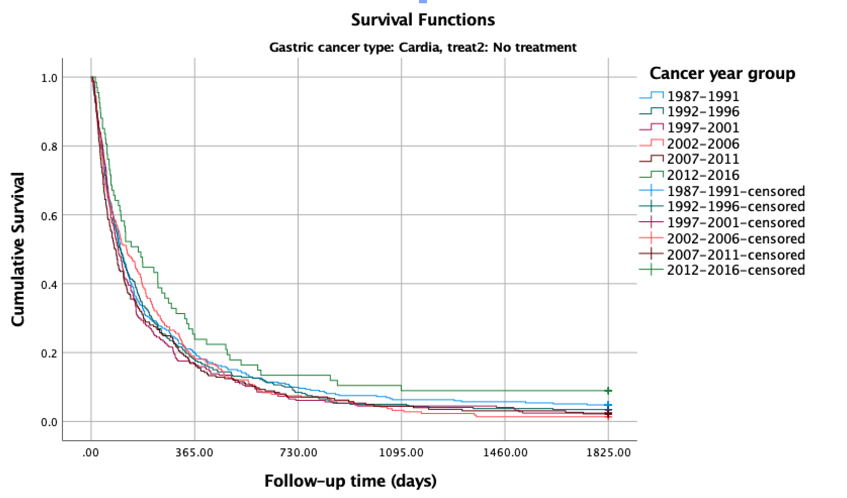
**

1. Cardia adenocarcinoma, no treatment

| 1987-1991 (n) | 332 | 66 | 33 | 21 | 19 | 15 |
| --- | --- | --- | --- | --- | --- | --- |
| 1992-1996 (n) | 319 | 57 | 27 | 16 | 12 | 10 |
| 1997- 2001 (n) | 245 | 41 | 15 | 11 | 10 | 5 |
| 2002-2006 (n) | 215 | 40 | 16 | 7 | 3 | 2 |
| 2007- 2011 (n) | 225 | 38 | 16 | 10 | 7 | 4 |
| 2012- 2016 (n) | 67 | 16 | 9 | 6 | 5 | 5 |

**Supplementary Figure 3:** Kaplan-Meier survival curves showing 5-year survival rate for histologically confirmed gastric non-cardia adenocarcinoma (a) and gastric cardia adenocarcinoma (b) undergoing surgery, for gastric non-cardia adenocarcinoma (c) and gastric cardia adenocarcinoma (d) undergoing chemotherapy only, and for gastric non-cardia adenocarcinoma (e) and gastric cardia adenocarcinoma (f) not undergoing treatment, stratified by 5-year time periods.


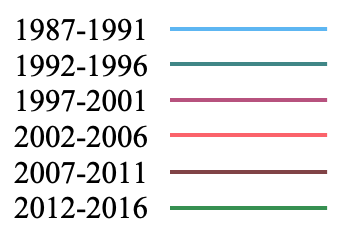

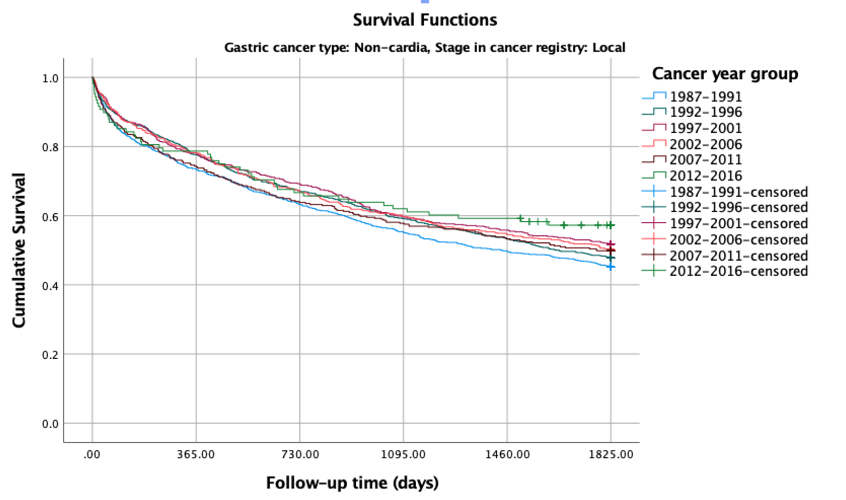


1. Non-cardia adenocarcinoma, local stage

| 1987-1991 (n) | 1092 | 802 | 691 | 604 | 543 | 493 |
| --- | --- | --- | --- | --- | --- | --- |
| 1992-1996 (n) | 960 | 745 | 644 | 568 | 512 | 458 |
| 1997- 2001 (n) | 609 | 476 | 421 | 363 | 340 | 314 |
| 2002-2006 (n) | 488 | 382 | 328 | 292 | 266 | 244 |
| 2007- 2011 (n) | 351 | 261 | 224 | 203 | 187 | 174 |
| 2012- 2016 (n) | 108 | 85 | 72 | 67 | 64 | 47 |


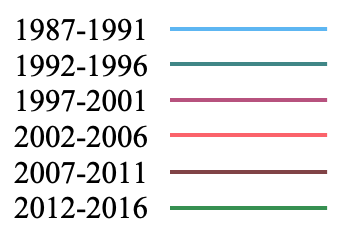

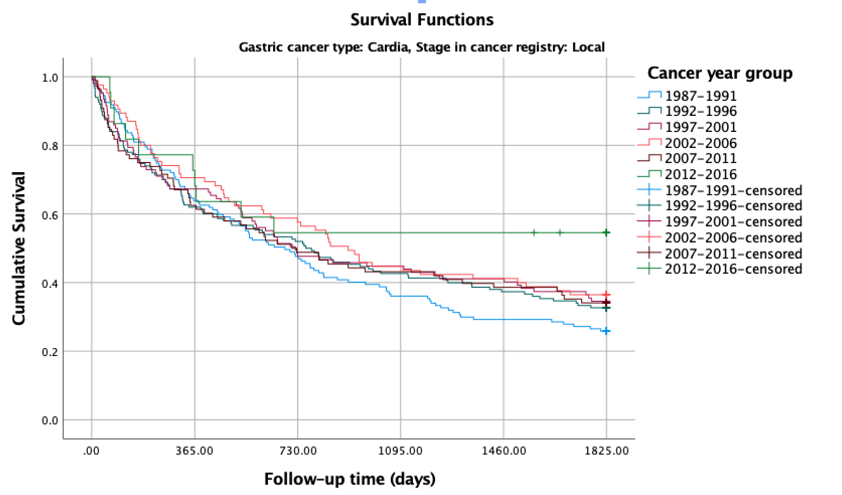


1. Cardia adenocarcinoma, local stage

| 1987-1991 (n) | 147 | 94 | 70 | 53 | 43 | 37 |
| --- | --- | --- | --- | --- | --- | --- |
| 1992-1996 (n) | 150 | 93 | 78 | 64 | 56 | 48 |
| 1997- 2001 (n) | 107 | 72 | 51 | 48 | 44 | 36 |
| 2002-2006 (n) | 85 | 60 | 49 | 38 | 35 | 30 |
| 2007- 2011 (n) | 88 | 55 | 43 | 38 | 34 | 29 |
| 2012- 2016 (n) | 22 | 15 | 12 | 12 | 12 | 9 |


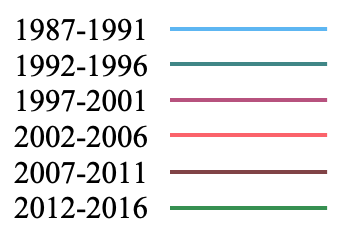

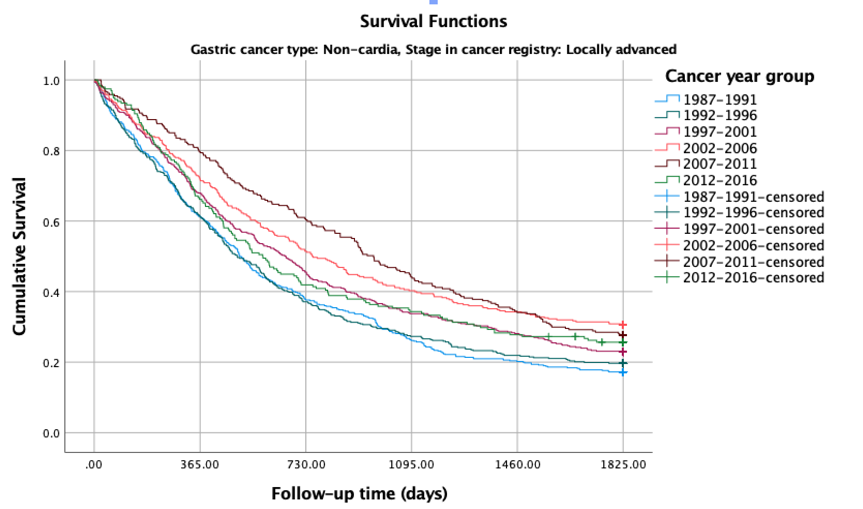


1. Non-cardia adenocarcinoma, locally advanced stage

| 1987-1991 (n) | 505 | 310 | 190 | 132 | 102 | 86 |
| --- | --- | --- | --- | --- | --- | --- |
| 1992-1996 (n) | 447 | 273 | 166 | 122 | 98 | 87 |
| 1997- 2001 (n) | 459 | 312 | 209 | 155 | 129 | 104 |
| 2002-2006 (n) | 350 | 251 | 180 | 141 | 120 | 106 |
| 2007- 2011 (n) | 267 | 212 | 161 | 118 | 91 | 73 |
| 2012- 2016 (n) | 198 | 131 | 83 | 68 | 55 | 45 |


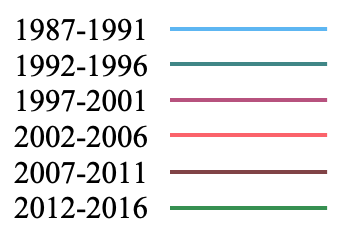

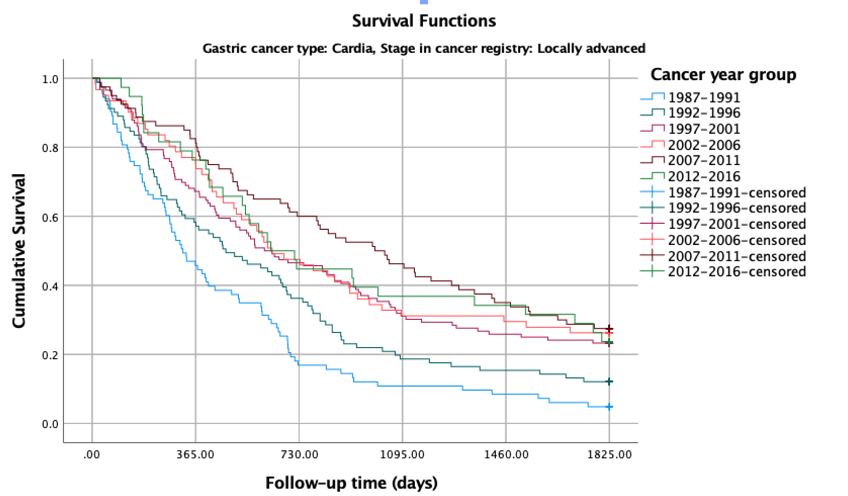


1. Cardia adenocarcinoma, locally advanced stage

| 1987-1991 (n) | 83 | 38 | 14 | 9 | 7 | 3 |
| --- | --- | --- | --- | --- | --- | --- |
| 1992-1996 (n) | 91 | 53 | 33 | 17 | 14 | 10 |
| 1997- 2001 (n) | 116 | 78 | 54 | 36 | 30 | 26 |
| 2002-2006 (n) | 61 | 46 | 29 | 19 | 18 | 15 |
| 2007- 2011 (n) | 80 | 65 | 48 | 37 | 28 | 21 |
| 2012- 2016 (n) | 38 | 29 | 17 | 14 | 13 | 8 |


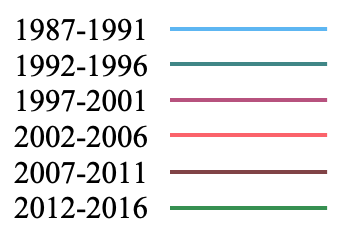

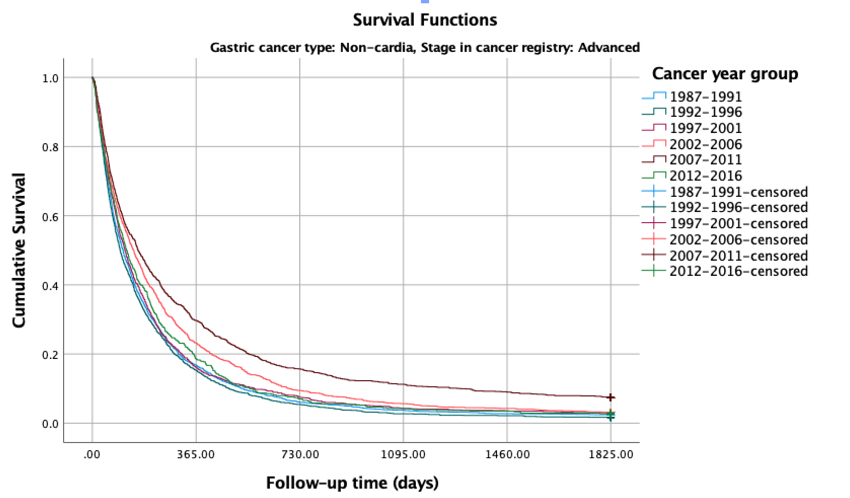


1. Non-cardia adenocarcinoma, advanced stage

| 1987-1991 (n) | 1892 | 318 | 114 | 71 | 53 | 44 |
| --- | --- | --- | --- | --- | --- | --- |
| 1992-1996 (n) | 1472 | 225 | 80 | 40 | 32 | 24 |
| 1997- 2001 (n) | 1203 | 195 | 90 | 51 | 43 | 36 |
| 2002-2006 (n) | 1286 | 299 | 122 | 73 | 55 | 39 |
| 2007- 2011 (n) | 1243 | 369 | 195 | 140 | 112 | 91 |
| 2012- 2016 (n) | 514 | 96 | 36 | 22 | 18 | 13 |


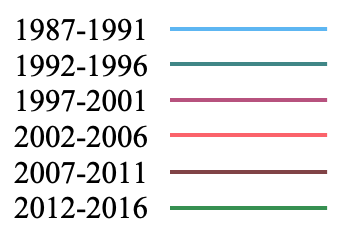

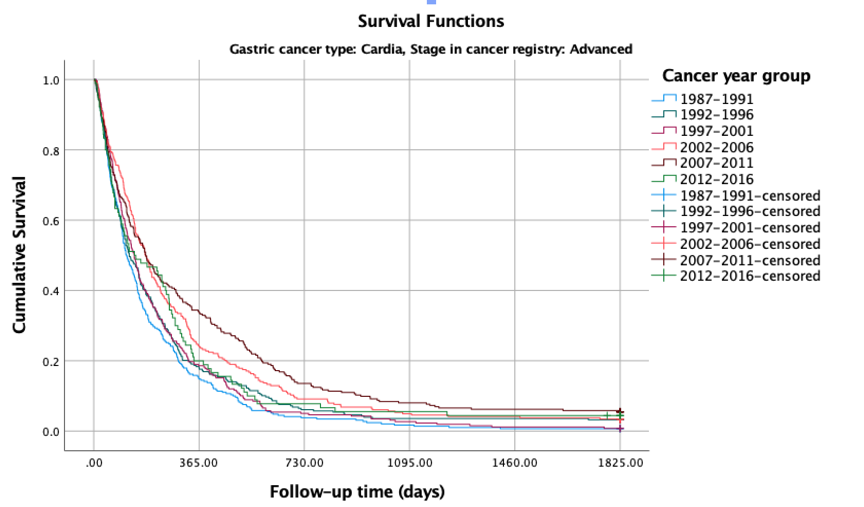


1. Cardia adenocarcinoma, advanced stage

| 1987-1991 (n) | 290 | 43 | 11 | 5 | 2 | 1 |
| --- | --- | --- | --- | --- | --- | --- |
| 1992-1996 (n) | 278 | 50 | 17 | 10 | 10 | 8 |
| 1997- 2001 (n) | 258 | 48 | 13 | 7 | 3 | 1 |
| 2002-2006 (n) | 263 | 64 | 24 | 13 | 11 | 8 |
| 2007- 2011 (n) | 273 | 92 | 37 | 22 | 17 | 14 |
| 2012- 2016 (n) | 90 | 18 | 7 | 5 | 4 | 2 |

**Supplementary Figure 4:** Kaplan-Meier survival curves showing 5-year survival rate for histologically confirmed gastric non-cardia adenocarcinoma (a) and gastric cardia adenocarcinoma (b) in local stage, for gastric non-cardia adenocarcinoma (c) and gastric cardia adenocarcinoma (d) in locally advanced stage, and for gastric non-cardia adenocarcinoma (e) and gastric cardia adenocarcinoma (f) in advanced stage, stratified by 5-year time periods.
